# Probing *Plasmodium falciparum* sexual commitment at the single-cell level

**DOI:** 10.12688/wellcomeopenres.14645.4

**Published:** 2018-10-17

**Authors:** Nicolas M.B. Brancucci, Mariana De Niz, Timothy J. Straub, Deepali Ravel, Lauriane Sollelis, Bruce W. Birren, Till S. Voss, Daniel E. Neafsey, Matthias Marti

**Affiliations:** 1 Wellcome Centre for Molecular Parasitology, Institute of Infection, Immunity & Inflammation, College of Medical, Veterinary and Life Sciences, University of Glasgow, Glasgow, UK; 2 Department of Immunology and Infectious Diseases, Harvard T.H. Chan School of Public Health, Boston, Massachusetts, USA; 3 The Broad Institute of MIT and Harvard, Cambridge, Massachusetts, USA; 4 Department of Medical Parasitology and Infection Biology, Swiss Tropical and Public Health Institute, Basel, Switzerland; 5 University of Basel, Basel, Switzerland

**Keywords:** Plasmodium falciparum, single cell, RNAseq, phenotyping, malaria, transmission, sexual differentiation

## Abstract

**Background:** Malaria parasites go through major transitions during their complex life cycle, yet the underlying differentiation pathways remain obscure. Here we apply single cell transcriptomics to unravel the program inducing sexual differentiation in *Plasmodium falciparum*. Parasites have to make this essential life-cycle decision in preparation for human-to-mosquito transmission.

**Methods:** By combining transcriptional profiling with quantitative imaging and genetics, we defined a transcriptional signature in sexually committed cells.

**Results:** We found this transcriptional signature to be distinct from general changes in parasite metabolism that can be observed in response to commitment-inducing conditions.

**Conclusions:** This proof-of-concept study provides a template to capture transcriptional diversity in parasite populations containing complex mixtures of different life-cycle stages and developmental programs, with important implications for our understanding of parasite biology and the ongoing malaria elimination campaign.

## Introduction

Malaria remains a major global health issue, with roughly 200 million infections and more than 400,000 fatal cases caused by *Plasmodium falciparum* each year ^1^. Despite decades of disease-control and elimination efforts, *P. falciparum* persists in many geographic regions, highlighting the adaptability of this parasite to changing environments. High levels of antigenic diversity have compromised efforts to develop efficacious vaccines, and resistance has evolved to all licensed antimalarial drugs ^2, 3^. Malaria parasites have a complex life cycle, and patient blood samples usually contain a mixture of asexually replicating parasites and a small fraction of terminally differentiated sexual-stage parasites. The latter, so-called mature gametocytes are required for parasite transmission to mosquitos. Blood-stage parasite isolates from malaria patients, or from resistance selection or *in vitro* culture adaption experiments, show significant heterogeneity in the transcriptional profile in population-level expression analyses ^4– 6^. To date, the transcriptional diversity of such mixed populations has not been captured appropriately, owing to lack of efficient single-cell mRNA profiling methods in *Plasmodium*. In this study, we present a cost-effective and high-throughput pipeline for single-cell transcriptional and phenotypic analysis of *P. falciparum* parasites. We used a digital gene expression (DGE) protocol ^7^ to define the transcriptional signature during initiation of parasite sexual differentiation (i.e. sexual commitment) and correlated mRNA profiles with microscopy-based phenotyping. Our study provides a template for capturing transcriptional diversity in heterogeneous parasite populations, which we hope will springboard future endeavors in single cell transcriptomics of *Plasmodium*.

To ensure transmission, malaria parasites invest in the production of gametocytes. During each intra-erythrocytic developmental cycle, a subset of asexually replicating parasites commits to produce sexual progeny ^8^. Rates of sexual commitment generally vary between species and conditions, and range from 1–30% in *P. falciparum* ^9^. Previous work suggested that one asexual parasite can produce either strictly sexual or strictly asexual progeny (i.e., be sexually or asexually committed) ^10^; however, conclusive evidence is lacking and the transcriptional profile of the sexually committed parasite has been poorly defined ^11, 12^. Two studies have identified AP2-G as a transcription factor that is activated during sexual commitment and required for gametocyte formation ^13, 14^. Initial analysis of gene expression in individual sexually committed cells using conditional AP2-G knock-down provided a first transcriptional signature upon AP2-G activation ^15^. We have recently demonstrated that availability of the host-derived phospholipid lysophosphatidylcholine (LysoPC) acts as an environmental sensor for the regulation of sexual commitment in *P. falciparum* ^16^. We have shown that LysoPC is the major building block for phosphatidylcholine biosynthesis in the parasite, and its depletion results in the induction of a compensatory metabolic response and triggers AP2-G activation and sexual commitment in a large part of the parasite population. This observation was facilitated through development of a highly controlled and quantitative *in vitro* assay to induce gametocyte formation ^16, 17^. Here we apply this assay to define the transcriptional signature of individual cells at different stages during sexual commitment and validate key findings experimentally.

## Results

### Development of a single-cell RNA-sequencing (scRNA-seq) pipeline in *P. falciparum*

**Figure 1.**
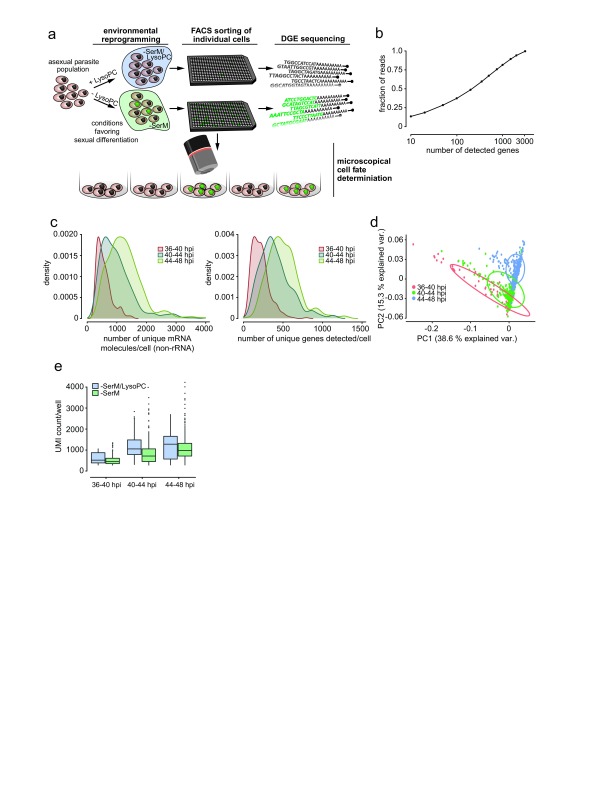
A digital gene expression (DGE) platform for *P. falciparum* single-cell mRNA profiling. ( **a**) Sexual commitment and experimental setup. Synchronized parasites of the Pf2004/164tdTom line are split into 2 populations, one exposed to inducing conditions using lysophosphatidylcholine (LysoPC)-deficient medium (–SerM) and one exposed to -SerM supplemented with LysoPC. Individual cells from each population are sorted by FACS for subsequent scRNAseq analysis. The remainder of cells from both populations is maintained in culture for measurement of parasite multiplication rate and sexual conversion. Sexually committed cells are denoted in green. ( **b**) DGE read counts per gene, sorted by highest to lowest expression in DGE. Approximately 200 genes account for 50% of all DGE reads. ( **c**) Distribution of DGE transcript counts (left panel) and genes detected (right panel) per cell, colored by time point. Density units are arbitrary but reflect the number of cells at a given value in the distribution. As read counts differed among and between time points, they were normalized per time point per cell prior to further analysis. ( **d**) Principal component analysis shows that single cell transcriptomes cluster according to time point. ( **e**) Unique mRNA transcripts per cell across time points are significantly lower in those incubated in SerM compared to those in –SerM supplemented with LysoPC.

To capture the transcriptional profile of sexual commitment, cells were exposed to a pulse of defined medium lacking LysoPC (−SerM), as described previously ^16^. At three time points after LysoPC depletion, 336 individual cells (and 48 control cells in the presence of LysoPC; −SerM/LysoPC) were collected by flow sorting and snap-frozen prior to being processed for DGE ( Figure 1a and Supplementary Figure 1). We utilized DGE owing to its capacity to quantitatively profile relative expression levels of genes from large numbers of individual cells without investment in specialized hardware, for a total cost of approximately $30/cell. Reads representing the 3’ end of transcripts from each cell were aligned to the *P. falciparum* reference genome (PlasmoDB version 29) and filtered for unique molecular identifier (UMIs) to avoid repeat sampling of the same original RNA molecules, and ribosomal RNA (rRNA) species were removed ( Table 1 and Supplementary File 1). Across cells we detected 3110 genes of the ~4900 genes transcribed at some level in blood stage parasites ^18^, and a smaller gene set was represented by multiple reads in the majority of cells. We considered genes to be detected if they exhibited at least 15 UMIs among the 881 cells that met our minimum sample quality criteria (described in the Methods). The 500 most highly transcribed genes account for approximately 65% of UMIs across all cells, while the 100 most highly transcribed genes account for approximately 40% of the UMIs ( Figure 1b). We found the number of UMIs per cell to vary across the three time points analyzed due to variation in library quality ( Figure 1c and Supplementary Figure 2), but cells in the second and third time points exhibited an average of 841 and 1118 UMIs, respectively. Principal component analysis (PCA) of normalized UMIs from highly expressed genes clustered individual cells by time point ( Figure 1d), demonstrating that stage-specific differences in transcriptional profiles are detectable in single *P. falciparum* parasites. Comparison of single cell expression profiles across the three time points to a previously published conventional bulk transcriptomic time series ^19^ confirms that the stage-specific differences we observe are indicative of cell-cycle progression rather than batch effects (linear regression, R ^2^ = 0.43; p < 2.2×10 ^-16^). Comparison of transcriptional profiles across time points revealed significantly reduced UMIs observed in cells grown under −SerM conditions compared to control (Wilcoxon rank sum, p = 0.007) ( Figure 1e), likely reflecting the reduced merozoite numbers we previously observed under these conditions ^16^. Next, we compared the transcription levels per gene across single cells with those from the same time points from a population-level RNA-seq experiment ^16^. The comparison demonstrated that overall expression levels per gene are significantly, though weakly, correlated between single-cell DGE and population-level RNA-seq (F-test, p<2.2×10 ^-16^) Notably, the weak correlation we observe between the two transcriptional datasets highlights the known gene “drop-out” effect of single-cell sequencing ^20, 21^. Altogether, these experiments demonstrate that the DGE platform presented here is able to capture mRNA profiles of single *P. falciparum* parasites at sufficient depth to i) detect transcriptional differences between 4-hour time points in the cell cycle and ii) recapitulate overall transcriptional profiles from population-level RNA-seq experiments.

**Table 1.** Mean values per cell. Mean read counts, UMIs and detectable genes per cell at each step in the processing pipeline shown for each time point.

Time point, hpi	Assigned reads	Aligned reads	UMIs	UMIs after excluding rRNA	Genes detectable
36-40	962,253	142,730	7,648	377	212
40-44	1,395,384	292,130	19,069	841	404
44-48	1,128,064	416,588	27,002	1,118	503

UMI, unique molecular identifier.

### Defining the transcriptional signature of sexual commitment

**Figure 2.**
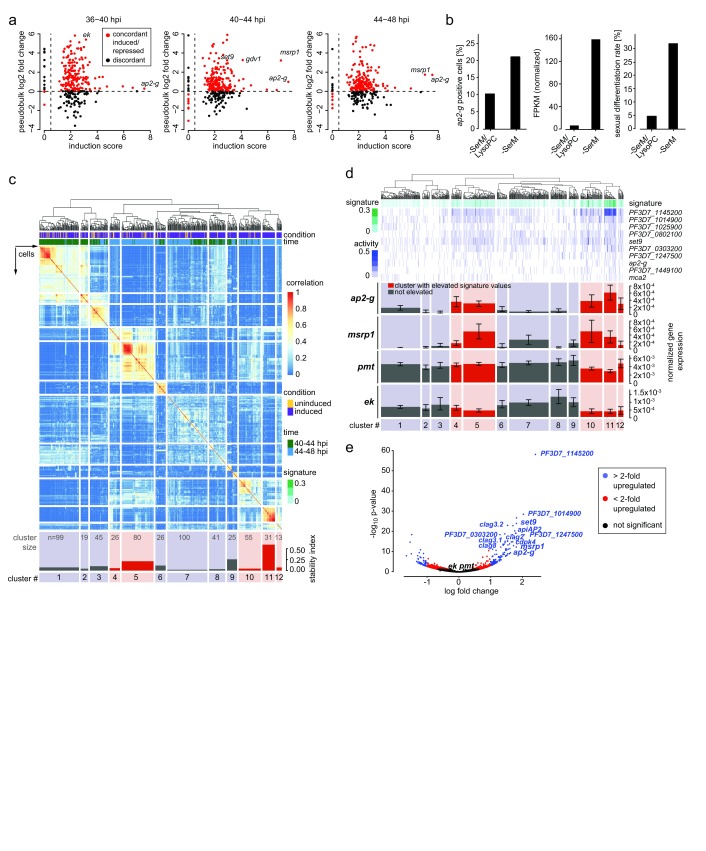
The transcriptional signature of sexually committed cells. ( **a**) Concordance of differential expression between pooled digital gene expression (DGE) reads (pseudobulk, shown as log _2_ fold change) and population-level RNA-seq (defined as log _2_ sum of the ratios of fragments per kilobase of transcript per million mapped reads in inducing over non-inducing conditions, or induction score ^16^). ( **b**) Comparison of *ap2-g* expression in single cells by DGE (left panel) and in population-level RNA-seq at 44-48 hpi (center). Sexual differentiation rates of cells used for DGE sequencing are shown in the right panel. ( **c**) Single-cell consensus clustering (SC3) defines a signature of sexual commitment. The subset of genes that were significantly differentially expressed from population-level RNA-seq was used to perform consensus clustering on the single cell RNA-seq gene expression. On top, a dendrogram showing k-means clustering results of single cells from 40-44 and 44-48 hpi. Just below, two parameters are shown: i) condition - induced cells are demarcated in purple and uninduced cells are demarcated in yellow; ii) time - cells from 40-44 hpi are in green and those from 44-48 hpi in blue. The rows and columns of the heat map are single cells that have been grouped into 12 clusters on the basis of their expression profiles (coloration indicating strength of clustering). The stability index (bottom) indicates how often those cells clustered together using different k values. Time point and condition from which the cells were derived are indicated, as well as positive expression of several genes of interest. Because *pmt* was highly expressed in most cells, only those with 80 ^th^ percentile or higher expression are marked. Cluster 11 revealed cells with high *ap2-g* expression and cluster stability. Cluster size is indicated. ( **d**) Transcriptional signature of commitment is enriched in five cellular clusters. Marker genes for SC3 cluster 11 were used to define a transcriptional signature of sexual commitment (see methods). This signature is a metric built on averaging normalized marker gene expression associated with cluster 11 (higher signature values are indicated with darker shades of green. The signature is shown in green above the heat map containing each gene’s individual expression values. Below, mean expression values and the standard error for *ap2-g*, *msrp1*, *pmt*, and *ek* are shown for each cluster. Clusters highlighted in red had significantly elevated signature values. Expression of *ap2-g* and *msrp1* is significantly enriched in the clusters highlighted in red, while expression of *pmt* and *ek* are not. ( **e**) Sexually committed cells show increased expression of 125 genes. Single cells from 40-48 hpi separate into subsets with high and low marker signature (above and below the 80 ^th^ percentile, respectively), with 125 differentially expressed genes measured by scRNA-seq, including *ap2-g*, *msrp1* and *set9*. Plotted *p* values reflect Benjamini-Hochberg correction.

LysoPC depletion induces sexual commitment in 25–35% of parasites across strains, while the remaining cells continue asexual replication ^16^. In the population-level experiment, we previously identified a set of 342 genes that were up-regulated upon LysoPC depletion, including the known commitment markers *ap2-g* ^13, 14^ and *gdv1* ^22^. Using the single cell mRNA profiles, we observed overall concordance in the induction signature when compared to the bulk RNA-seq data ( Figure 2a and Supplementary File 2). The increase of cells with detectable *ap2-g* transcripts upon LysoPC depletion ( Figure 2b) with the proportion of sexual progeny in the same experiment, confirming that *ap2-g* activation in a cell is predictive of its differentiation state. Considering the mixed commitment of parasites to either the sexual or the asexual pathway under inducing conditions *in vitro* ^17^, the transcriptional profile from our population-level RNA-seq experiment likely represented a combination of different transcriptional signatures. Specifically, we hypothesized that the changes induced by LysoPC depletion represent both a general response in all cells as well as a sexual commitment-specific activation of *ap2-g* and other factors required to initiate sexual differentiation in a subset of cells. Single-cell consensus clustering (SC3) ^23^ based on gene expression profiles from 40–48 hpi revealed robust and reproducible separation of individual cells into 12 clusters ( Figure 2c, Supplementary Figure 3, Supplementary Figure 4, and Supplementary File 3). Detectable or elevated expression of 10 genes ( Figure 2d), including *ap2-g* and *set9*, defined a subset of cells (cluster 11), presumably representing the minimal transcriptional signature of sexually committed cells. This signature was significantly enriched in 36% of all cells (205/560; Wilcoxon rank sum test, corrected p<0.05) comprising five cell clusters (4, 5, 10, 11, and 12; Figure 2d). A total of 125 genes were significantly co-expressed with this transcriptional signature, including several kinases, epigenetic regulators, transcription factors and multiple resident rhoptry proteins ( Figure 2e and Supplementary Figure 4). By contrast, ethanolamine kinase ( *ek*) and phosphoethanolamine methyl transferase ( *pmt*), two key enzymes that are induced upon LysoPC depletion ^16^ and needed for phosphatidylcholine biosynthesis under these sexual commitment-inducing conditions, were equally expressed across all cells ( Figure 2d), rather than co-expressed with this transcriptional signature ( Figure 2e).

Comparative analysis of our single-cell data with those from Poran *et al.* ^15^ and the bulk RNA-seq dataset validated the results by revealing a significant overlap in genes with commitment-associated expression profiles, despite differences in experimental setup and sequencing pipelines ( Table 2 and Supplementary File 5). Poran *et al.* ^15^ also observed an enrichment of AP2-G binding motifs upstream of commitment-associated genes. We observe a similar enrichment of genes with upstream AP2-G binding sites within 2 kb of the translational start site (as defined in Campbell *et al.* ^24^) within our commitment signature gene set (117 out of 125; *p* = 0.002, Fishers exact test, indicating that a significant proportion of the loci we detected may be downstream of AP2-G activation. Altogether, single-cell transcriptional profiling demonstrated that LysoPC depletion induces both a population-wide transcriptional response and a sexual commitment-specific response in a subset of cells.

**Table 2.** Comparison of hits between the three studies. These are results from the R package SuperExactTest, which statistically tests if there is an enrichment of overlapping genes in multiple sets ^25^. The expected overlap is the null hypothesis that there is only random sampling of genes. The p-value is the one-tail probability of observing equal or larger overlapping genes at random. The background size was set to 5,800, which is how many transcripts there are in v29 of the 3D7 transcriptome. For comparison, we used hits from Brancucci *et al*. (bulk) ^16^, this study (scRNA-seq data in Supplementary File 4, all hits with positive fold change in last two time points; sc) and Poran *et al.* ^15^ (scRNA-seq data in table S3, all hits; Poran).

Intersections	Degree	Observed overlap	Expected overlap	FE	*p*-value
Brancucci *et al.* ^16^ (bulk; Supplementary File 2, TP3/4)	1	268			
Poran *et al.* ^15^ (Table S3)	1	28			
Brancucci *et al.* (sc; Supplementary File 4 + FC)	1	125			
Poran & bulk	2	9	1.29	6.96	2.67E-06
Poran & sc	2	3	0.60	4.97	0.022
sc & bulk	2	14	5.78	2.42	0.0017
Poran & sc & bulk	3	2	0.03	71.73	3.64E-04

Fe, fold enrichment over null; FC, fold change; sc, single cell.

### Schizonts produce either asexual or sexual progeny

**Figure 3.**
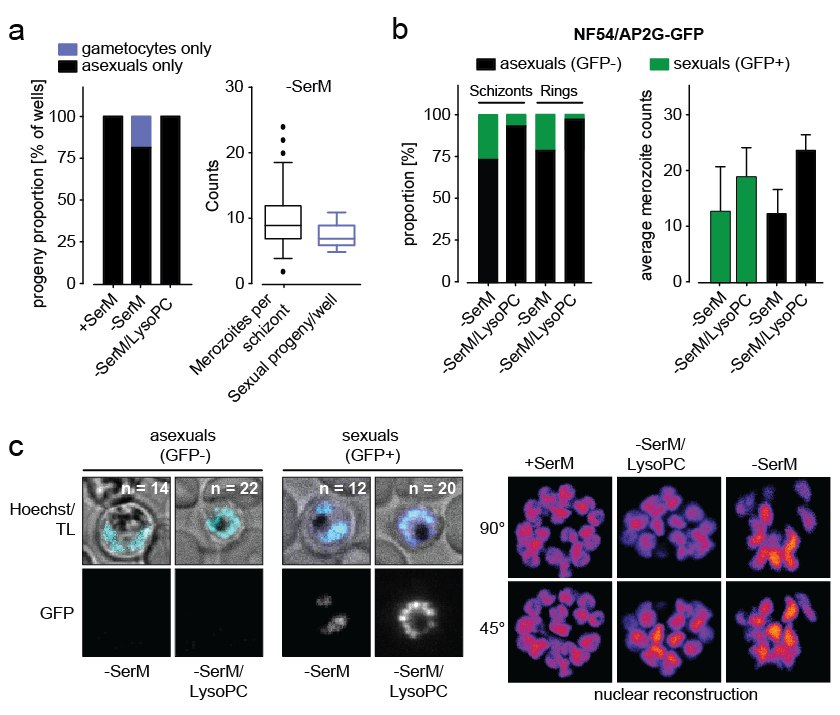
Progeny analysis in single cells. ( **a**) Analysis of progeny from 96 single cells in the Pf2004/164tdTom reporter line (expressing tdTomato reporter under the gametocyte-specific *PF10_0164* promoter ^17^). Each parasite produces only asexual or gametocyte progeny (left panel). The number of sexual progeny corresponds to the number of daughter merozoites per schizont under lysophosphatidylcholine-deficient medium (−SerM) conditions (right panel). A total of 50 independent measurements were made per condition. ( **b**) Analysis of sexually committed schizonts in the NF54/AP2-G GFP reporter line. The proportion of GFP positive schizonts and the number of GFP positive progeny (rings) correlate and mark sexual cells (gametocyte rings). Both sexually committed (GFP-positive) and asexually committed schizonts show reduced merozoite number under −SerM conditions (right bar graph). GFP-positivity was quantified in 100`000 cells by ImageStream analysis in biological triplicates. Merozoite counts represent the mean of 100 measurements per condition, performed in biological triplicates. ( **c**) Representative images from cells in ( **b**) and selected planes from 3D reconstructions of nuclei are shown in the left and right panels, respectively. Hoechst, nucleic acid stain; TL, transmitted light. n = number of nuclei.

Identification of a distinct transcriptional signature in AP2-G-expressing (AP2-G+) cells supports the hypothesis that sexual and asexual progeny are non-uniformly distributed among schizonts. This hypothesis is also reinforced by the recent scRNA-seq study ^15^, and by previous work using plaque assays and progeny analysis with gametocyte-specific antibodies ^10^. To validate these observations directly, we investigated sexual commitment in reporter parasites. Parasites were grown in −SerM and −SerM/LysoPC conditions and separated into individual wells for further development. Analysis of progeny from single schizonts confirmed the absence of mixed differentiation states, with progeny numbers corresponding to the number of merozoites per parental schizont ( Figure 3a). Moreover, the prevalence of reporter-positive schizonts corresponded to the number of positive progeny (i.e., sexual rings) in the same culture upon LysoPC depletion ( Figure 3b). Notably, merozoite numbers were similarly reduced in both sexually committed and asexually committed schizonts in −SerM medium compared to control ( Figure 3b, c). These data support the observation of a population-wide transcriptional response, in agreement with the transcriptional activation of *ek* and *pmt* in all cells under −SerM conditions ( Figure 2e).

### Experimental validation of induction and sexual commitment signatures

**Figure 4.**
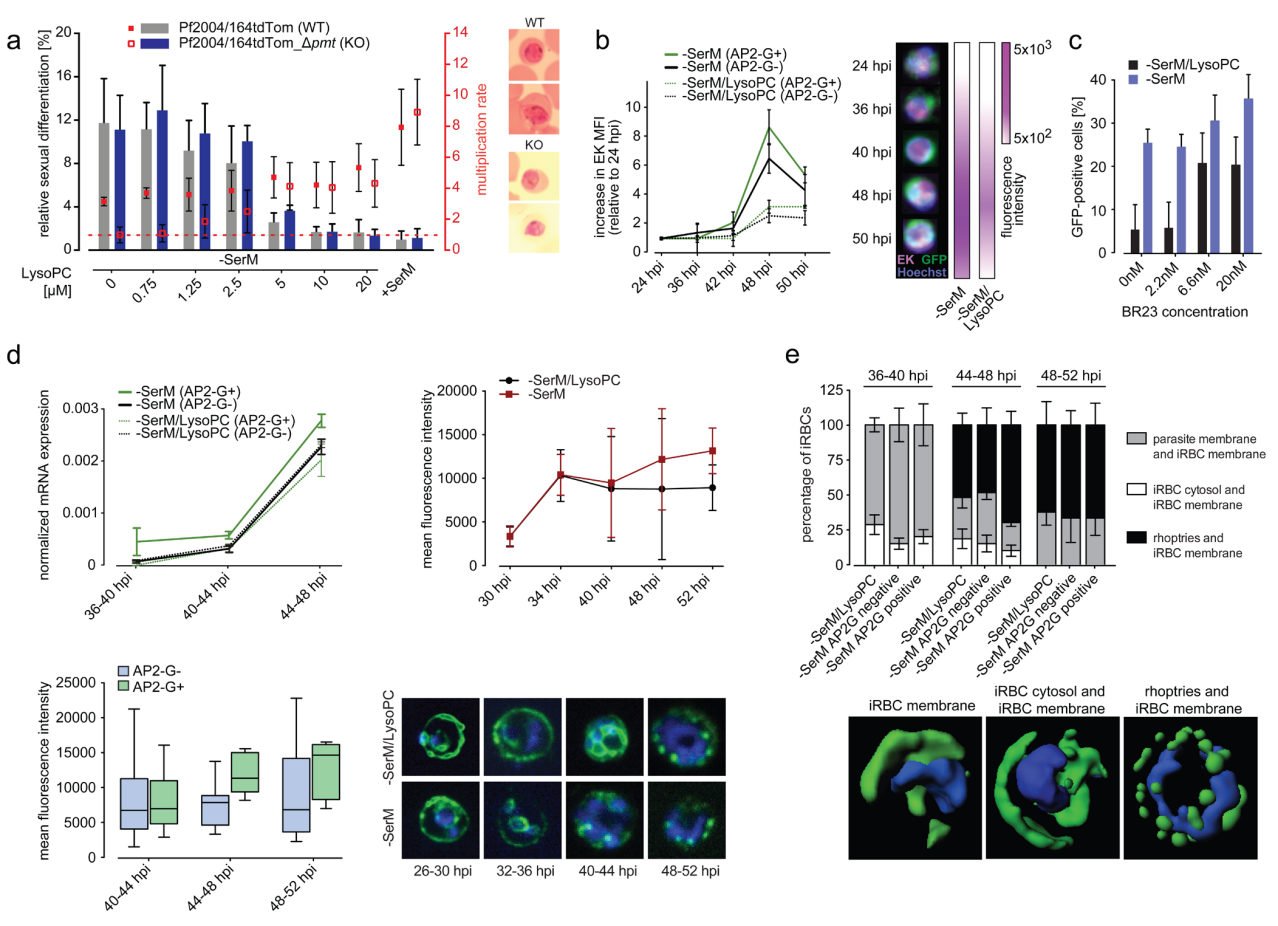
Experimental validation of the single-cell RNA-seq signature. ( **a**) Phosphoethanolamine methyl transferase (PMT) is not required to trigger sexual commitment. *pmt* knock-out parasites (Pf2004/164tdTom_Δ *pmt*) induce sexual commitment in response to lysophosphatidylcholine (LysoPC) depletion at rates that are similar to those of control parasites (left axis). By contrast, asexual growth is significantly impaired in the knock out cell line under LysoPC limiting conditions (right axis). Differences in parasite multiplication increase with lower lipid concentrations, demonstrating that *pmt* activity becomes essential for the compensatory metabolic response induced at low LysoPC levels. The horizontal dashed line indicates complete growth inhibition (multiplication rate of 1). Right panel: Giemsa smears of parasites at the time point of gametocyte readout. No difference in morphology between WT and KO parasite is observed at 0 μM LysoPC, suggesting normal gametocyte development until Stage II. ( **b**) Analysis of EK expression in the NF54/AP2-G GFP reporter line. EK expression increases significantly over time and is induced under LysoPC-deficient medium (−SerM) conditions. Cells were stained with anti-EK antibody (far red), anti-GFP antibody (green) and the nuclear dye Hoechst 33342 (blue). All parasites (AP2-G+ and AP2-G-) show increased EK expression under −SerM conditions. Representative composite images are shown. Data in the left panel are the result of 100,000 measurements performed by ImageStream in biological duplicate experiments with technical triplicates. Error bars represent interquartile ranges. ( **c**) Choline kinase inhibition activates AP2-G expression. Specific inhibition of choline kinase by BR23 induces sexual commitment at growth-limiting concentrations (IC50 of BR23: 3.5 nM). ( **d**) Rhoptry gene expression is increased in AP2-G expressing cells. The average normalized gene expression from DGE was calculated for all rhoptry-related genes. Cells expressing AP2-G have significantly higher levels of overall rhoptry gene transcription (p=0.001), even when accounting for time, LysoPC treatment, and raw transcript counts (upper left panel). RhopH3 expression is increased during sexual commitment compared to control. Time course analysis comparing RhopH3 expression between −SerM and −SerM/LysoPC control (upper right panel), and between AP2-G+ and AP2-G- (lower left panel; *p* = 0.002 for time point 2 and *p* = 0.01 when all time points are pooled). Representative images per time point and condition are shown (lower right panel). The number of cells analysed was 100, per time point per condition. ( **e**) RhopH3 localization in –SerM/LysoPC and –SerM treated cells, at 36, 44 and 48 hpi. Localization dynamics were analyzed for rhoptries, infected red blood cell (iRBC) cytosol and iRBC membrane. Time point 2 shows significant differences when comparing AP2-G+ and AP2-G- cells ( *p*=0.001 for rhoptries and iRBC membrane; *p*=0.004 for parasite membrane and iRBC membrane). Rendered representative images of the three main localizations are shown. The number of cells analysed was 100, per time point per condition.

To test whether PMT activity is essential in absence of LysoPC (in all cells, irrespective of their differentiation state) and required for sexual commitment, we generated *pmt* knock-out parasites using the CRSIPR/Cas9 technology. Genetic disruption of *pmt* demonstrated that parasites lacking PMT activity are unable to grow in the absence of LysoPC, while growth is unaffected under control conditions. By contrast, sexual commitment levels were not altered in *pmt* knock-out parasites, demonstrating that activation of sexual commitment is independent of the compensatory activation of this metabolic enzyme under LysoPC-limiting conditions ( Figure 4a and Supplementary Figure 5, Supplementary Figure 6) ^16^. In support of these data, imaging flow cytometry also revealed increased protein expression of the second enzyme involved in producing choline from the alternative substrate ethanolamine (ethanolamine kinase, *ek*) in all cells under −SerM conditions ( Figure 4b). Confirming the data obtained from the SC3 approach described above, these results show that while EK and PMT are induced upon LysoPC depletion, activation of these enzymes is not specific to the pathway inducing sexual commitment. By contrast, we observed a titratable increase in AP2-G+ cells by inhibition of the essential Kennedy pathway enzyme choline kinase using the specific inhibitor BR23 ^16, 26^ ( Figure 4c and Supplementary Figure 6), indicating that a metabolite or enzymatic activity downstream of this reaction translates the LysoPC signal into a cellular response favoring asexual replication over sexual commitment.

As stated above, we also observed enrichment of multiple rhoptry markers in our sexual commitment signature ( Figure 2d, e), including *RhopH1/2/3* and several rhoptry neck proteins. Rhoptry genes were generally transcribed at higher levels in all AP2-G+ cells across all three time points, even when accounting for time point and –SerM conditions (t=4.7, p=3.1×10 ^-6^) ( Figure 4d). In agreement with these transcriptional data, analysis of RhopH3 protein expression over time by immunofluorescence microscopy confirmed significantly increased levels in sexually committed cells (AP2-G+) compared to AP2-G- cells and control conditions ( Figure 4d). RhopH3 is present in rhoptries and required for merozoite invasion into red blood cells (RBC), and at the infected RBC surface as part of a nutrient channel ^27^. The protein is initially localized to rhoptries and upon invasion it is translocated into the host cell cytosol and surface membrane. Our image analysis demonstrated significantly increased RhopH3 localization in rhoptries but not in the host cell in AP2-G+ cells compared to AP2-G-, confirming that *de novo* synthesis of this protein is elevated in sexually committed schizonts ( Figure 4e). Altogether these data validate the findings from the SC3 analysis and highlight the complex nature of the parasite response to changes in LysoPC availability.

## Discussion

We have applied scRNA-seq to investigate the signature of sexual commitment in *P. falciparum* malaria parasites, with particular focus on the events that precede the activation of the transcriptional master switch AP2-G.

We have recently identified the host phospholipid LysoPC as a natural repressor of commitment, and LysoPC depletion as a means to induce AP2-G activation *in vitro* ^16^. Combining this assay with scRNA-seq has revealed a transcriptional signature in sexually committed cells. As expected, there was only modest (but significant) overlap with the signature defined by Poran *et al.*, as their experiment was not designed to profile commitment-associated processes upstream of AP2-G activation. Apart from *ap2-g*, the only commonly activated gene detected by both studies was the merozoite antigen *msrp1* ^28, 29^. *msrp1* has an AP2-G-binding site in its upstream sequence ^15^, suggesting that it is activated by (and therefore downstream of) AP2-G. We observed significant overlap between our scRNA-seq and the population RNA-seq data ^16^, as the same experimental set-up was used in these two studies. Shared hits include two kinases, a histone methyl transferase and two additional ApiAP2 transcription factors, further supporting the hypothesis that intracellular signaling events translate nutrient availability into epigenetic changes that ultimately regulate AP2-G. Using a combination of scRNA-seq, quantitative imaging of single cells and reverse genetics we demonstrate that the compensatory activation of *ek* and *pmt* is a general response to nutrient depletion and independent of parasite sexual commitment. Our work also demonstrates that PMT is essential for asexual growth in absence of LysoPC (or choline) as a substrate for PC synthesis. This finding reiterates the compensatory metabolic role of PMT under LysoPC-limiting conditions. By contrast, inhibition of the first enzyme in the Kennedy pathway, choline kinase, using the specific inhibitor BR23 activates AP2-G expression, even in the presence of LysoPC. These data suggest that a metabolite or enzymatic activity in the Kennedy pathway downstream of choline kinase is necessary to repress AP2-G activation. LysoPC uptake is drastically reduced in gametocytes ^16^, suggesting that PMT activation is both a general response to nutrient depletion and preparation for increased requirement of this alternative substrate arm during gametocyte development. Indeed, previous work has demonstrated that PMT is essential for gametocyte maturation and mosquito infection ^30, 31^.

Malaria parasites are exposed to changing environments during their life cycle, and these changes usually coincide with developmental switches. In addition, blood-stage parasites have to adapt to constantly changing nutrient availability. Recent work has demonstrated that the major energy source for the parasite, glucose, is used as an environmental sensor to modulate progeny number and hence virulence via a signaling cascade ^32^. Surprisingly, no effect on transmission was observed under caloric restriction, and glucose complementation did not block sexual conversion in our study ^16^. These findings suggest the existence of several environmental sensing pathways in the parasite that regulate parasite growth, transmission and possibly other phenotypes, such as immune evasion via antigenic variation. The pathways must be finely tuned in order to ensure an optimal balance between growth and transmission, rather than a population-level shift towards one state. We hypothesize that the decision for commitment is regulated through a bistable biological switch reinforced through a positive feedback loop, such as the known auto-activation of AP2-G ^13, 15^ and possibly involves kinase-mediated signaling upstream of this transcription factor. In such a scenario, individual cells may show stochastic variation in the threshold concentration of LysoPC required to induce commitment and activate AP2-G. Such threshold-dependent regulation may explain why sexual commitment rates in *P. falciparum* are generally confined to upper limits (i.e., below 50%). While commitment rates can be elevated beyond these limits in response to genetic modifications introduced under *in vitro* conditions ^11, 15^, limiting them in response to environmental triggers will ensure sufficient levels of asexual growth within the human host as a prerequisite for transmission success ^9, 33^.

Our data also demonstrate that blood stage schizonts harbor only sexual or asexual merozoites, but not mixed progeny. Moreover, we noted the enrichment of a number of rhoptry-associated proteins in the sexual commitment signature and were able to validate this observation experimentally with RhopH3 on the protein level. Quantitative imaging revealed increased RhopH3 expression in rhoptries, specifically in sexually committed schizonts. Identification of different or differentially expressed sets of merozoite antigens, including *rhoph3* and *msrp1*, in sexual versus asexual merozoites suggests that these two populations may be functionally distinct. Such differences may relate to altered host cell tropism and/or the emerging model of a specific tissue niche for gametocyte formation and development ^34– 37^. Sexual commitment rates are generally low and elevated levels of invasion markers may also increase the likelihood of successful invasion and therefore transmission. The concept of distinct merozoite populations is supported by the variegated expression of some invasion ligands ^38^ and the recent identification of differences between hepatic and blood stage merozoites ^39^.

In summary, our study is one of the first single-cell transcriptomic studies of *Plasmodium* parasites, and applies this approach to define the signature of parasite commitment to the sexual pathway. Complementary chemical and genetic experiments validate the transcriptional data and define the different roles of the Kennedy pathway and its alternative substrate arm in growth and sexual commitment. Together with the recent studies by Poran *et al*. ^15^ and Reid *et al.* ^40^, our scRNA-seq approach introduces methodology that provides a blueprint to investigate key knowledge gaps in the complex parasite cycle that are driven by transcriptional variation across cells. As such, these studies also represent powerful tools in addition to the available genetic and phenotypic assays in order to track parasite behavior during the ongoing malaria elimination campaign.

## Methods

### Generation of CRISPR/Cas9 pmt knock-out cell line

To disrupt the Pf *pmt* gene (PF3D7_1343000) in the Pf2004/164tdTom cell line, we generated the transfection vector pB_gC-Pf *pmt*. The pB_gC Cas9/sgRNA mother plasmid contains expression cassettes for (i) the *Streptococcus pyogenes* Cas9 enzyme, (ii) the single guide RNA (sgRNA), and (iii) the BSD resistance marker flanked by homology boxes ^16^. To generate pB_gC-Pf *pmt*, two complementary oligonucleotides (sgRNA_ *pmt_*F, sgRNA_ *pmt*_R) encoding the sgRNA target sequence for *pmt* and appropriate single-stranded overhangs were annealed and inserted into the sgRNA expression cassette using *Bsa*I-digested pB_gC and In-Fusion cloning. The *pmt* sgRNA target sequence (ATATTCTTCAACAGTTATTA) is positioned in the coding sequence 254 bp upstream of the stop codon. The donor cassette was generated using In-Fusion ® HD cloning kit (Takara) to join PCR fragments *pmt* homology region 1 amplified from Pf2004_164Tdtom gDNA using primers pmt_HR1_F and pmt_HR1_R, and *pmt* homology region 2 generated using primers pmt_HR2_F and pmt_HR2_R. PCR was performed with Phusion HF DNA polymerase (NEB) with the following thermocycling conditions: initial denaturation at 95°C for 2 min, thermocycling with denaturation at 95°C for 30 sec, annealing gradient ramp from at 50°C to 58°C for 30 sec, elongation at 61°C for 1 min for 8 cycles, 22 additional cycles [denaturation at 95°C for 30 sec, annealing at 57°C for 30 sec, elongation at 61°C for 1 min] and final elongation step at 61°C for 8 min. In the edited strain (Pf2004/164tdTom *-*Δ *pmt*), integration of *bsd* resistance marker was verified by PCR using primers *pmt*_5’UTR_F and BSD_5’UTR_R primer (testing 5’ integration) and BSD_3’UTR_F and *pmt*_3’UTR_R (testing 3’ integration). PCR conditions were as follows: initial denaturation at 95°C for 2 min, thermocycling with denaturation at 95°C for 30 sec, annealing at 51°C for 30 sec, and elongation at 61°C for 1 min for 25 cycles, and final elongation step at 61°C for 8 min. All oligonucleotide sequences are provided in Supplementary File 6.

### Parasite culture

*P. falciparum* cell culture was performed as described previously ^17, 41^. Culture media consisted of RPMI-1640 supplemented with 25 mM HEPES, 100 μM hypoxanthine, 24 mM sodium bicarbonate, and gentamicin (all from Sigma-Aldrich). Serum media was generated by additional supplementation of 10% O+ human serum (The Interstate Companies), while serum-free medium was generated by additional supplementation of 0.39% fatty acid-free BSA, 30 μM oleic acid, and 30 μM palmitic acid (all from Sigma-Aldrich). To generate serum-free media supplemented with 20 μM LysoPC, LysoPC (Avanti Polar Lipids) was dissolved in ethanol and dried on culture dishes prior to culture addition. All cell cultures were maintained in a 5% CO _2_, 1% O _2_ and 94 % N _2_ gas mixture (Med-Tech Gases). All experiments used a clone of the Pf2004/164TdTomato *P. falciparum* line ^17^, which requires addition of 4 nM WR99210 (Jacobus Pharmaceuticals) to select for stable maintenance of episomes.

### Parasite transfection and selection of transgenic parasites

Pf2004/164tdTom ring stage parasites were transfected with 100 µg pB_gC-Pf *pmt* plasmid using electroporation conditions as described ^42^. To select for parasites carrying the a disrupted *pmt* locus (Pf2004/164tdTom_Δ *pmt*), transfected parasites were grown in presence of 2.5 µg/ml blasticidin-S deaminase for the first 10 days and then in absence of drug pressure until a stably propagating parasite population was established (approximately 4 weeks post-transfection). Successful disruption of the *pmt* gene in the 164tdTom_Δ *pmt* population was confirmed by PCR on gDNA DNeasy blood and tissue kit (Qiagen), with thermocycling and primers, as described under “Generation of CRISPR/Cas9 *pmt* knock-out cell line”. All oligonucleotide sequences are provided in Supplementary File 6.

### Gametocyte induction and sample collection

Parasite cultures of the Pf2004/164tdTom line were sorbitol-synchronized (Sigma-Aldrich) as described previously ^43^ to a 4-hour cycle window using four consecutive treatments across two growth cycles. At 28–32 hours post-invasion, parasites were washed in −SerM media and plated with or without LysoPC. At 4, 8, and 12 hours post induction, cells were stained with Vybrant DyeCycle Violet (Life Technologies) in HBSS (Thermo Fisher Scientific). Stained cells were pelleted and resuspended in phenol red-free PBS supplemented with 0.3% RNaseOUT (Life Technologies). Collection Twin.tec PCR 384-well plates (Eppendorf) were prepared by addition of 5 µl of cell lysis buffer to each well; lysis buffer consisted of nuclease-free water (Thermo Fisher Scientific) supplemented with 0.2% HF buffer (New England Biolabs). Single Vybrant Dye Cycle Violet-positive cells were sorted using a Beckman Coulter MoFlo Astrios EQ. At each time point, 48 cells from the non-induced culture (+LysoPC) and 336 cells from the induced culture (-LysoPC) were sorted into the collection plate and snap frozen on dry ice. Cultures were maintained for an additional two cycles for parasitemia and gametocytemia measurements. The media of both non-induced and induced cultures was replaced with RPMI/serum 12 hours post-induction and daily thereafter.

### Conversion rate measurement

To measure parasitemia, samples were stained with SYBR Green (Life Technologies) and measured by flow cytometry using a MACSQuant Analyzer 10 (Miltenyi Biotec). To measure gametocytemia, samples were again stained with SYBR Green and measured by flow cytometry using the BioRad S3. Gametocyte conversion rate was calculated as described previously ^17^.

### Sequencing

We used an optimized version of single-cell RNA barcoding and sequencing ^6^ (SCRB-seq; ^7^), with some modifications to enable high throughput automated liquid handling. Briefly, cells were thawed on ice and each sample was lysed using 1 µl of 1 mg/ml Proteinase K (ThermoFisher Scientific) followed by incubation at 50°C for 15 min, then 95°C for 10 min uncovered to evaporate all liquid. Reverse transcription was performed in 2 µl reactions containing 0.4 µl of 5X Maxima H Minus Reverse Transcriptase Buffer (ThermoScientific), 0.2 µl dNTPs (10 mM each; NEB), 0.02 µl of 100 µM E5V6NEXT primer (/5Me-isodC/iisodG/iMe-isodC/ACACTCTTTCCCTACACGACGCrGrGrG; IDT), 1 µl of 2 µM E3V6NEXT barcoded primer (see Soumillon *et al*. ^7^ for barcoded primer sequences) and 0.125 µl Maxima H Minus Reverse Transcriptase (200 U/µl) (Thermo Fisher Scientific). Reactions were incubated at 42°C for 90 minutes followed by incubation at 80°C for 10 minutes, then pooled together. Pooled cDNA was purified using a DNA Clean & Concentrator kit-5 (Zymo Research) according to the manufacturer’s recommended protocol for single-stranded DNA. Purified cDNA was then exonuclease-treated in a 20 µl reaction using 2 µl of 10X Exonuclease I Reaction Buffer (NEB) and 1 µl of Exonuclease I enzyme (NEB). Reactions were incubated at 37°C for 30 minutes, then at 80°C for 20 minutes. The cDNA was then amplified in a 50 µl reaction using the Advantage 2 PCR Kit (Clontech) as follows: 5 µl of 10X Advantage 2 PCR Buffer, 1 µl dNTPs, 1 µl of 10 µM SINGV6 primer (/5Biosg/ACACTCTTTCCCTACACGACGC) (IDT) and 1 µl Advantage 2 Polymerase Mix (Clontech) using the following cycling program: 95°C, 1 min; 18 cycles of 95°C, 15 sec, 65°C, 30 sec, 68°C 6 min; 72°C 10 min. Reactions were purified using AMPure XP beads (Beckman Coulter) according to the manufacturer’s recommended protocol. Illumina sequencing libraries were prepared using 1 ng of purified cDNA as input material for the Nextera XT kit (Illumina) according to the manufacturer’s recommended protocol, with the exception of using the P5NEXTPT5 primer (AATGATACGGCGACCACCGAGATCTACACTCTTTCCCTACACGACGCTCTTCC*G*A*T*C*T) (IDT) instead of the standard i5 primers contained in the kit. Final libraries were purified using AMPure XP beads (Beckman Coulter) according to the manufacturer’s recommended protocol and prepared for sequencing on an Illumina NextSeq500 using paired reads of 17bp (library insert read1) + 46bp (library insert read2).

### DGE read processing

DGE reads have the following organization. Read1 of the pair contains a well barcode, which signifies which well in a 384-well plate a read came from, and a unique molecular index (UMI). The UMI is used to distinguish reads representing different transcripts from reads originating from PCR duplicates. Read2 contains 45 nucleotides originating from the 3’ end of an mRNA transcript.

Reads from each time point were processed as follows. Reads were assigned to a well using the well barcode, allowing for one mismatch between sequence and barcode (all barcodes are at least two nucleotides different from each other). Reads without valid UMIs were also discarded. Read sequences were aligned to the PlasmoDB *Plasmodium falciparum* 3D7 transcript (v29) downloaded on Oct 19, 2016 using bwa aln (v 0.7.10), allowing for up to four mismatches between the read and the reference. This mismatch-tolerance approach yielded more usable data than trimming ends of reads exhibiting a drop-off in base quality. Reads were also discarded if there were 20 or more As in a row (i.e., polyA tail) or if the sequence is mapped to more than 20 different transcripts. If there were duplicate UMIs, only one was counted. We aligned reads to the hg19 human genome using the same alignment parameters as above to evaluate whether reads that did not map to the *P. falciparum* reference transcriptome instead aligned to the human transcriptome, but observed an overall low alignment rate, suggesting that reads not assignable to *P. falciparum* most likely represent low quality and/or low complexity sequence rather than human contamination. To confirm our suspicion, we used FastQC (v 0.11.4) to assess sequence quality on aligned and unaligned reads to *P. falciparum*.

### Further downstream processing of read counts

Post-processing and statistical analyses were performed using R version 3.3.1. UMI counts for all ribosomal RNA transcripts were removed. Genes that were expressed in fewer than 20 wells across all three time points were also excluded, which removed 2,576 genes from the 5,686 non-rRNA genes, to give a total of 3,110 genes measured. Wells containing fewer than 300 filtered UMIs were then removed, leaving 191/384 wells from 36-40 hpi, 326/384 wells from 40-44 hpi, and 364/384 wells from 44-48 hpi. Following filtering and removal, each time point was normalized using edgeR (v 3.14.0), such that each well’s total normalized expression values summed to 1. The rRNA genes that were used for filtering are: *PF3D7_0112300*, *PF3D7_0112500*, *PF3D7_0112700*, *PF3D7_0531600*, *PF3D7_0531800*, *PF3D7_0532000*, *PF3D7_0725600*, *PF3D7_0725800*, *PF3D7_0726000*, *PF3D7_0801100*, *PF3D7_0801200*, *PF3D7_0830000*, *PF3D7_0830200*, *PF3D7_1148600*, *PF3D7_1148620*, *PF3D7_1148640*, *PF3D7_1371000*, *PF3D7_1371200*, *PF3D7_1371300*, *PF3D7_1418500*, *PF3D7_1418600*, *PF3D7_1418700*, *PF3D7_API04900*, *PF3D7_API05700*, *PF3D7_API05900*, *PF3D7_API06700*.

### Downstream analysis

***Principal component analysis (PCA).*** PCA was performed in R using the function prcomp, with center set to true and scale set to false (as data were already normalized). PCA was performed on edgeR-normalized read counts across all three time points (combined). PCA plots were visualized using the R package ggbiplot (v 0.55).

***Comparison of DGE expression profiles to Bozdech *et al.*^19^ expression profiles.*** Normalized expression profiles from 46 times points from 1-48 hpi at 1-hour intervals were obtained from supplementary data from Bozdech *et al*. ^19^. Only genes that were observed in both datasets were retained, and further filtered to the highest 100 expressed genes by DGE (though these results appeared to be robust to varying numbers of included genes). The Pearson correlation coefficient was obtained for normalized gene expression from each individual cell to each individual time point described by Bozdech *et al*. ^19^. Then, each cell was assigned to the maximally correlated time point from the above study. A linear regression model was generated in R comparing the time (in hpi) the cell came from ( *i.e.* 38 42, 46 hpi) to the maximally correlated time (in hpi) in 19. Adjusted R-squared and p-values obtained from the F test are reported.

***Detection of differentially expressed genes.*** Since DGE read counts were sparse, we first pooled UMI counts from wells at random into three distinct replicates for induced and uninduced separately, for each time point, creating “pseudobulk” gene expression profiles. Then, to detect differentially expressed genes, edgeR was employed, comparing pseudobulk counts of induced vs uninduced, for each time point. Differential expression was determined using a general linear model likelihood ratio test (glmLRT). Log2 fold change and p-value are reported for each gene. Multiple hypothesis correction was done using the Benjamini & Hochberg method.

***SC3.*** The R package SC3 was used to perform single cell consensus clustering on normalized gene expression of those genes that were detected to be differentially expressed in the bulk RNA-seq experiment. The built in function using the Tracy-Widom theory on random matrices to estimate the optimal number of clusters *k* was used to pick the number of clusters. Cluster strength was determined by iterating over *k*-1 to *k*+1 clusters in SC3. The marker signature was calculated by averaging all marker genes’ expression from cluster 11 from SC3 with *k* equal to 12, normalizing genes across cells first, so as to give equal weight to all genes, regardless of how highly expressed they are. Enrichment of the marker signature was determined using one-vs-rest Wilcoxon rank sum test for each cluster with Benjamini & Hochberg correction.

***Other statistics.*** All statistics were performed in R. The p-values comparing *ap2-g* expression between inducing conditions as well as time points were calculated using a Fisher’s exact test on the contingency tables. Linear regression for population-level RNA-seq versus DGE expression was performed using a linear model (lm in R), with adjusted R ^2^ and p-values reported. Correlation coefficients and p-values for *ap2-g* versus other genes were calculated using a paired samples Pearson’s product moment correlation test (cor.test in R). The overlap between single-cell RNA-seq experiments was evaluated with the R package SuperExactTest (v 1.0.0), which calculates the size of intersections of multiple sets and evaluates their statistical significance, as described previously ^25^.

### Parasite live imaging and quantitative analysis of progeny numbers

Prior to imaging, *P. falciparum* trophozoite-infected RBCs were incubated in either of 3 different conditions, namely minimal fatty acid medium (−SerM), −SerM medium supplemented with 20 μM LysoPC (−SerM/LysoPC), or serum-complemented media (+SerM). Following 4–6 hour incubation in these inducing or non-inducing conditions, all parasites were incubated in RPMI/serum, and allowed to develop until early schizont (34-38 hpi), late schizont (40-48 hpi), or ring stages (48-54 hpi).

For merozoite quantification, late trophozoites/early schizonts were processed as described by Grüring *et al.* ^44^ and Brancucci *et al*. ^16^. Briefly, cells from either −SerM, −SerM/LysoPC, or +SerM culture were arrested on the glass bottom of a sterile, concanavalin A-coated dish. Concanavalin A (ConA; Sigma-Aldrich) was dissolved at a concentration of 0.5 mg per ml in dH _2_O, and 200 µl distributed uniformly on the glass surface of a 27 mm dish (Scientific Laboratory Supplies). ConA was added to the dish for 30 minutes at 37°C. It was then washed off twice using 1x PBS, after which the culture was re-suspended in PBS, added to the glass bottom of the dish, and allowed to settle for 10–20 minutes at 37°C. Thereafter, non-bound cells were washed off using PBS leaving a monolayer in the glass bottom, and 2 ml of pre-warmed PBS were added to the dish for imaging. Cells were viewed using a Zeiss Observer Z1 spinning disc confocal microscope equipped with an incubation chamber, a Yokogawa CSU-X1 filter wheel and spinning disc unit, a Photometrics Evolve 512 delta EM-CCD camera, two laser lines: 488nm, 405nm, and an α-Plan-Apochromat 100x 1.46 NA DIC VIS immersion oil lens. Parasites were immobilized in a glass-bottom dish as described above, stained with Hoechst 33342, and 50-72 z-stacks (0.2-μm step size) obtained using bright field and the 488 nm and 405 nm laser line, to determine the number of nuclei in RBCs infected with AP2G-GFP-positive or AP2G-GFP-negative parasites (when using the NF54 AP2G-GFP parasite line) or alternatively in all infected red blood cells of the Pf2004164tdTom parasite line. Quantifications were performed at either 2- or 4-hour intervals between 24 and 54 hpi.

### Collection of individual *P. falciparum* parasites and progeny analysis

Between 36 and 40 hpi parasites at a parasitemia of 2% were allowed to settle on a glass-bottomed dish at a hematocrit of 0.05%. This enabled capturing of single parasites by pipette aspiration. Using a 100x oil immersion objective, and DIC or BF illumination, parasites were identified by hemozoin crystal presence or schizont structure when possible. Parasites were transferred to an intermediate glass-bottomed dish, where the presence of a single parasite was confirmed, and the parasite was ultimately transferred to a single well of a 96-well plate (glass bottom) at 0.5 % hematocrit in complete RPMI. This was repeated for 96 parasites per condition within a 4-hour time window. In the 96-well plates, each schizont was able to successfully rupture, after which merozoites successfully invaded neighboring uninfected RBCs. Four days post-infection, Hoechst 33342 at a concentration of 1:1000 was added to each well, and the wells viewed using a Zeiss Observer Z1 (specifications described above). Two proxies were analyzed using a 40x water objective, namely number of nuclei detected per well (using a 405 nm laser line), and number of TdTomato-positive cells per well (using a 594 nm laser line), the latter being exclusive to gametocyte progeny. The total number of TdTomato-positive cells and Hoechst-positive cells per well was quantified.

### Parasite fixed imaging and quantitative analysis of AP2-G, ethanolamine kinase and RhopH3

For imaging of AP2-G and ethanolamine kinase levels in fixed cells at various times post-infection under −SerM and −SerM/LysoPC conditions, parasites were collected from culture at 24, 36, 40, 42, 44, 48, and 50 hpi, washed 3 times with 1x PBS, and fixed in 4% paraformaldehyde/0.01% glutaraldehyde in PBS for 1 hour, followed by permeabilization using 0.1% Triton X for 10 minutes. Parasites were then washed and blocked in 3% BSA overnight. All samples were incubated for 1.5 hours with a mouse anti-ethanolamine kinase antibody (dilution 1:100) ^45^, and a polyclonal rabbit anti-GFP antibody (dilution 1:200, Abcam #ab6556) diluted in 1 % FBS in PBS. After 3 washes in PBS with 1 % FBS, AlexaFluor488 monoclonal anti-rabbit IgG (1:500, Thermo Fisher #A27034), and AlexaFluor647 monoclonal anti-mouse IgG (1:500, BD Biosciences #557681) was added to the cells and incubated for 30 min. After 3 additional washes, the nuclear dye Hoechst 33342 diluted 1:1000 in Hank’s Balanced Salt Solution (Sigma-Aldrich) was added for 30 minutes at RT.

RhopH3 was used as previously published ^27^, namely using a 1:1 ratio of acetone:methanol, followed by blocking in 3% milk powder. The RhopH3 rabbit antibody was diluted 1:500 and incubated for 40 min at room temperature. For detection, either AlexaFluor647 or AlexaFluor488 anti-rabbit antibodies were used at a dilution of 1:1000 for 30 minutes at room temperature.

 Samples were examined using a Zeiss observer Z1 spinning disc confocal microscope, equipped with a Yokogawa CSU-X1 filter wheel and spinning disc unit, a Photometrics Evolve 512 delta EM-CCD camera, three laser lines (405, 488, and/or 642 nm) and an α-Plan-Apochromat 100x 1.46 NA DIC VIS immersion oil lens. Images were collected in line sequential mode with a dwell time of 1.1 µs and z-increments of 0.19 µm. Additionally, images were acquired using an ImageStream X Mark II imaging flow cytometer, using a 40x objective. Images were acquired using Zen 2012 software, and processed using ImageJ (version 2.0.0-rc-43/1.51r), Ideas (version 4.0), or Imaris software (version 8.2.0). For quantitation of rhoptry localization, surface rendering and segmentation was performed using Imaris, and further analysis was done using ImageJ.

## Data availability

All raw data from experimental procedures (imaging and flow cytometry experiments) are available on OSF under project title “Probing *Plasmodium falciparum* sexual commitment at the single cell level”: https://doi.org/10.17605/OSF.IO/QMXVZ ^46^. Data are available under the terms of the Creative Commons Zero "No rights reserved" data waiver (CC0 1.0 Public domain dedication). The .czi (Carl Zeiss imaging files) and .lif (Leica imaging files) files can be accessed using the Bio-Formats plugin for ImageJ; the .fcs (flow cytometry input files) and .wps files can be accessed using FlowJo.

Data from expression profiling by high-throughput sequencing of the *Plasmodium falciparum* NF54 line, GSE96066. http://identifiers.org/geo:GSE96066.

## Supplementary material

**Supplementary Figure 1. DNA content of collected samples.** At each time point, cells were VybrantViolet-stained to label (parasite) DNA. Plots show VybrantViolet intensity for infected cells from each sample. Lysophosphatidylcholine (LysoPC)-deficient medium (−SerM) is shown on the left; −SerM supplemented with LysoPC is shown on the right.

**Supplementary Figure 2. Quality of reads across time points and aligned vs unaligned.** FastQC analysis reveals that aligned reads from 36-40 hpi are of lower quality, thus accounting for decreased number of UMI counts in that time point. Unaligned reads across all three time points have substantially lower-quality scores, likely explaining why these reads could not be aligned. In addition, unaligned reads are enriched in polyA tails, as exemplified by the increase in %A at the 3' end. (a) Per position base quality scores for each time point, separated by aligned vs unaligned reads. (b) Per read mean base quality for each time point, separated by aligned vs unaligned reads. (c) Per base sequence content of unaligned reads.

**Supplementary Figure 3. Single-cell consensus clustering (SC3) based on all filtered cells from 36-48 hpi.** (a) Expression correlation across all cells by SC3. The dendrogram shows separation of cells into 12 clusters. Positive expression of *set9* (dark blue), *pmt* (80 ^th^ percentile, orange), *msrp1* (cyan), and *ap2-g* (magenta) is indicated *.* The stability index of each cluster is given (bottom). (b) Marker genes of cluster #6 from SC3 of 36-48 hpi cells. The heat map shows transcriptional activity, with higher (red), moderate (yellow), and low (blue) levels. The top seven marker genes in cluster #6 are identical to those detected in cluster #11 from SC3 of 40-48 hpi.

**Supplementary Figure 4. Expression dynamics of commitment markers**. ( **a**) Life cycle expression dynamics of single-cell consensus clustering (SC3) marker genes. Normalized gene expression of marker genes from SC3 cluster #11 per time point from 1-48 hpi based on Bozdech *et al*. ^19^. Only marker genes observed in 19 are shown. Expression of these marker genes peaks around 38 hpi. ( **b**) Life cycle expression dynamics of rhoptry marker genes. All rhoptry-related genes from Bozdech *et al*. ^19^ are shown. The majority of these genes are also highly expressed after 36 hpi, with a peak around 39 hpi.

**Supplementary Figure 5. Cloning strategy for Pf *pmt* knock out.** To disrupt the Pf *pmt* gene (PF3D7_1343000) in parasites, we generated the CRISPR/Cas9 transfection vector pB_gC-Pf *pmt*. This vector was obtained by inserting a *bsd* resistance cassette flanked by the 5’ and 3’ homology regions (HR) into the p_gC mother plasmid ^16^. ( **a**), Schematic maps of the wild-type *pmt* locus in Pf2004/164tdTom parasites (top), the pB_gC-Pf *pmt* transfection vector (center), and the disrupted *pmt* locus after CRISPR/Cas9-based integration of the *bsd* resistance marker (bottom). The position of the *pmt* sgRNA target sequence 254 bp upstream of the *stop* codon is indicated. The pB_gC- *pmt* Cas9/sgRNA plasmid contains expression cassettes for Cas9, the sgRNA, and two donor homology regions ( *5’HR* and *3’HR*) flanking the *bsd* resistance marker facilitate repair of the Cas9-induced double-strand break and deletion of *pmt* gene by double crossover homologous recombination. Blue and red arrows indicate positions of PCR primer binding sites used to confirm successful gene editing. ( **b**) PCR on gDNA isolated from Pf2004/164tdTom_Δ *pmt* parasites and Pf2004 wild-type control parasites. Primers *pmt_5`UTR_F* and *pmt_3`UTR_F* bind to wild-type sequences outside the *pmt* 5’HR and *ap2-g* 3’HR homology regions and amplify a 2148 bp fragment from the wild type *pmt* locus (note the edited locus did not yield a product due to the large fragment size). The *pmt*_5’UTR_F/BSD_5’UTR_R and BSD_3’UTR_F/ *pmt*_3’UTR_R primer combinations are specific for the edited locus in Pf2004/164tdTom_Δ *pmt* parasites and amplify 957 and 861 bp fragments, respectively.

**Supplementary Figure 6. Growth phenotypes of parasites with impaired Kennedy pathway metabolism.** ( **a**) *pmt* knock-out parasites (Pf2004/164tdTom_Δ *pmt*) show reduced growth compared to control parasites (Pf2004/164tdTom) under lysophosphatidylcholine (LysoPC)-limiting conditions only. The horizontal line indicates complete inhibition of growth (multiplication rate of 1). ( **b**) LysoPC depletion shifts inhibitory concentration of BR23 (IC _50_ −SerM: 1.42 nM, 95% CI 1.23-1.64; IC _50_ −SerM/LysoPC: 3.54 nM, 95% CI 2.9-4.66). BR23 efficiently inhibits growth in presence of LysoPC, demonstrating the essential role of its target choline kinase. In absence of LysoPC, parasites become even more sensitive to BR23 suggesting that this drug also blocks the compensatory response through inhibition of the reported EK activity of choline kinase ^26^.

**Supplementary File 1. Normalized read counts and unique molecular identifiers (UMIs) across single cells.**

**Supplementary File 2. Significantly differentially expressed genes in single cells under −SerM conditions.**

**Supplementary File 3. Cluster membership and marker gene results from single cell consensus clustering (SC3) analysis.**

**Supplementary File 4. Genes differentially expressed in cells with a marker signature above the 80 ^th^ percentile across 40-44 and 44-48 hpi.**

**Supplementary File 5. Overlapping hits between the three studies.** Shown are all 22 hits that overlap between at least two out of the three studies. AP2-G and MSRP1 are the only hits overlapping in all three studies, confirming that AP2-G expression represents a major commitment signal. The small set of overlapping hits likely defines a minimal machinery driving the sexual commitment pathway. This core set includes several transcriptional and cell cycle regulators, as we well as signaling molecules but no metabolic enzymes, confirming our experimental data.

**Supplementary File 6. Oligonucleotides used in this study.**
