# Supplementary figures and images for: Probing Plasmodium falciparum sexual commitment at the single-cell level
Source: Wellcome Open Res. 2018 Oct 17;3:70. Originally published 2018 Jun 13. [Version 4] doi: 10.12688/wellcomeopenres.14645.4 (PMC6143928; doi:10.12688/wellcomeopenres.14645.4)

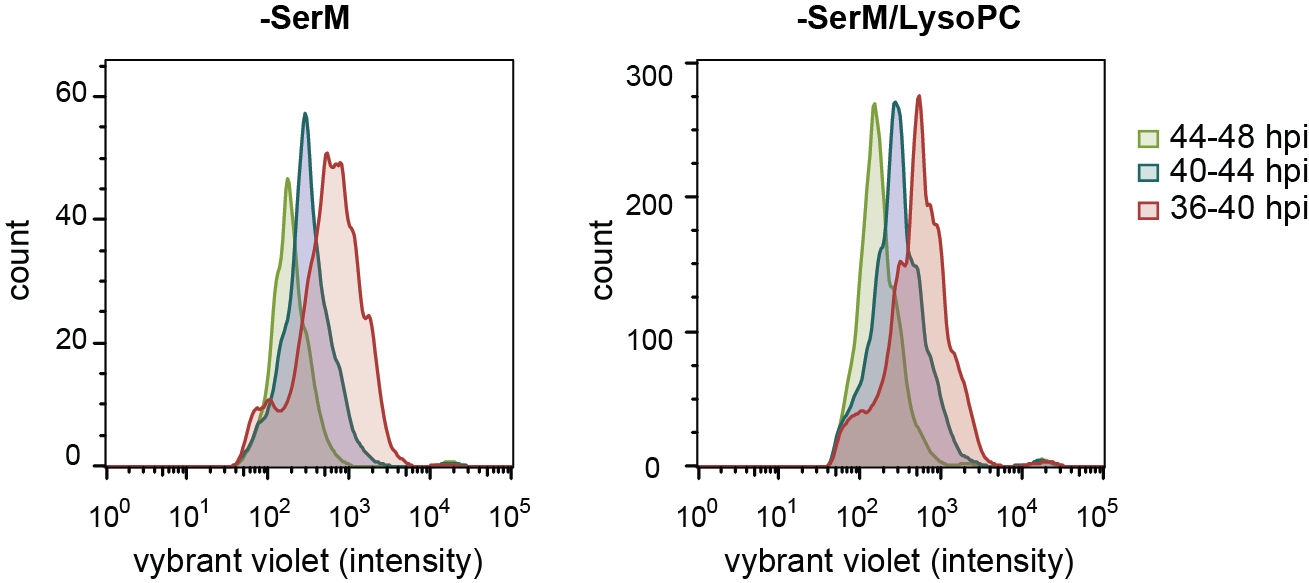

Supplement: Supplementary file 1 [file wellcomeopenres-3-16217-s0000.tgz › 2d06add1-4298-4e54-ac19-3cfe067b1712.png]

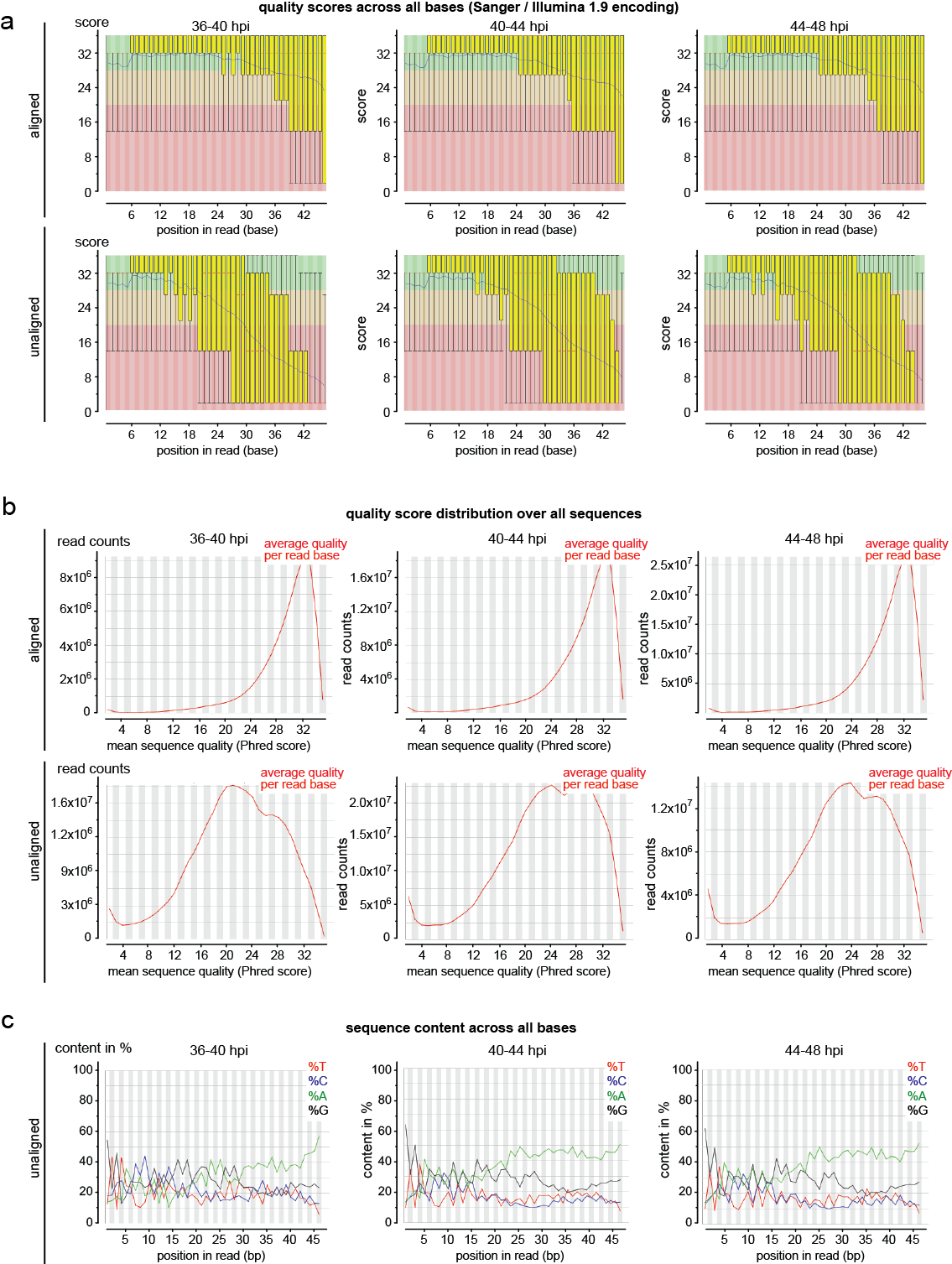

Supplement: Supplementary file 2 [file wellcomeopenres-3-16217-s0001.tgz › f2a15f08-50f4-47ea-8dfa-73e845bb319f.png]

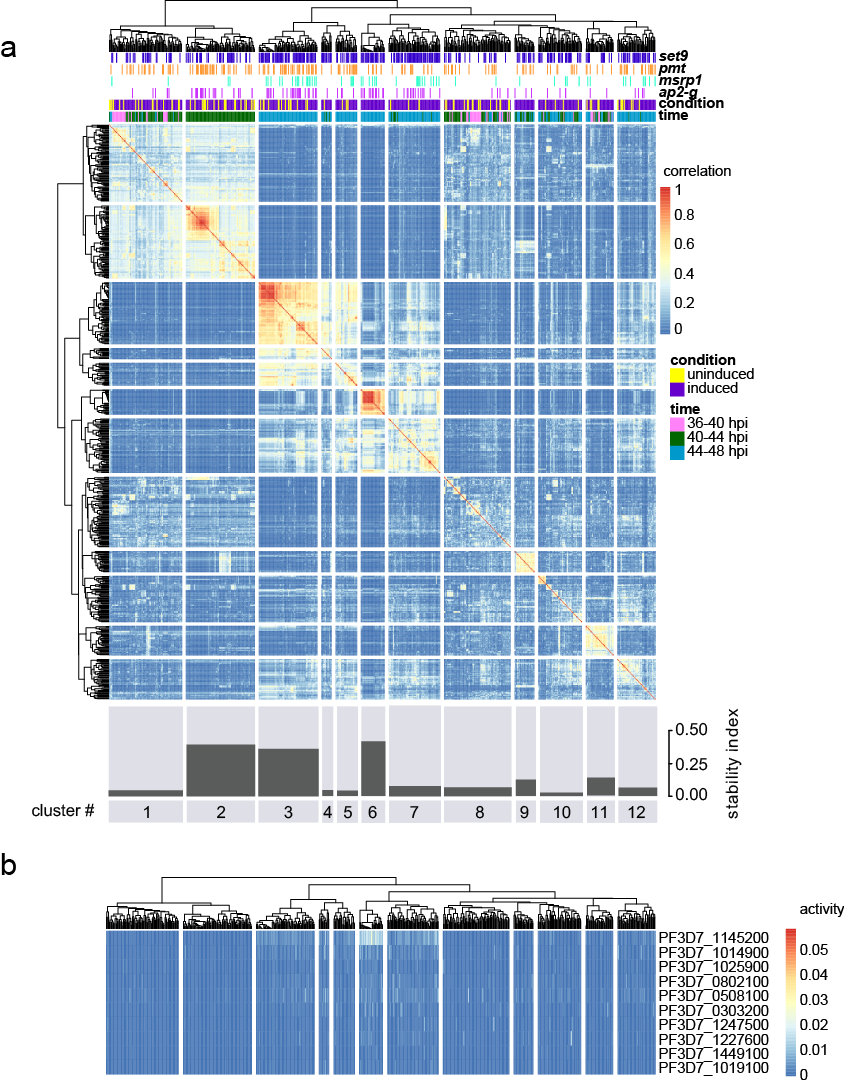

Supplement: Supplementary file 3 [file wellcomeopenres-3-16217-s0002.tgz › 57cf6a40-95fb-469b-a9c8-d770eaee4138.png]

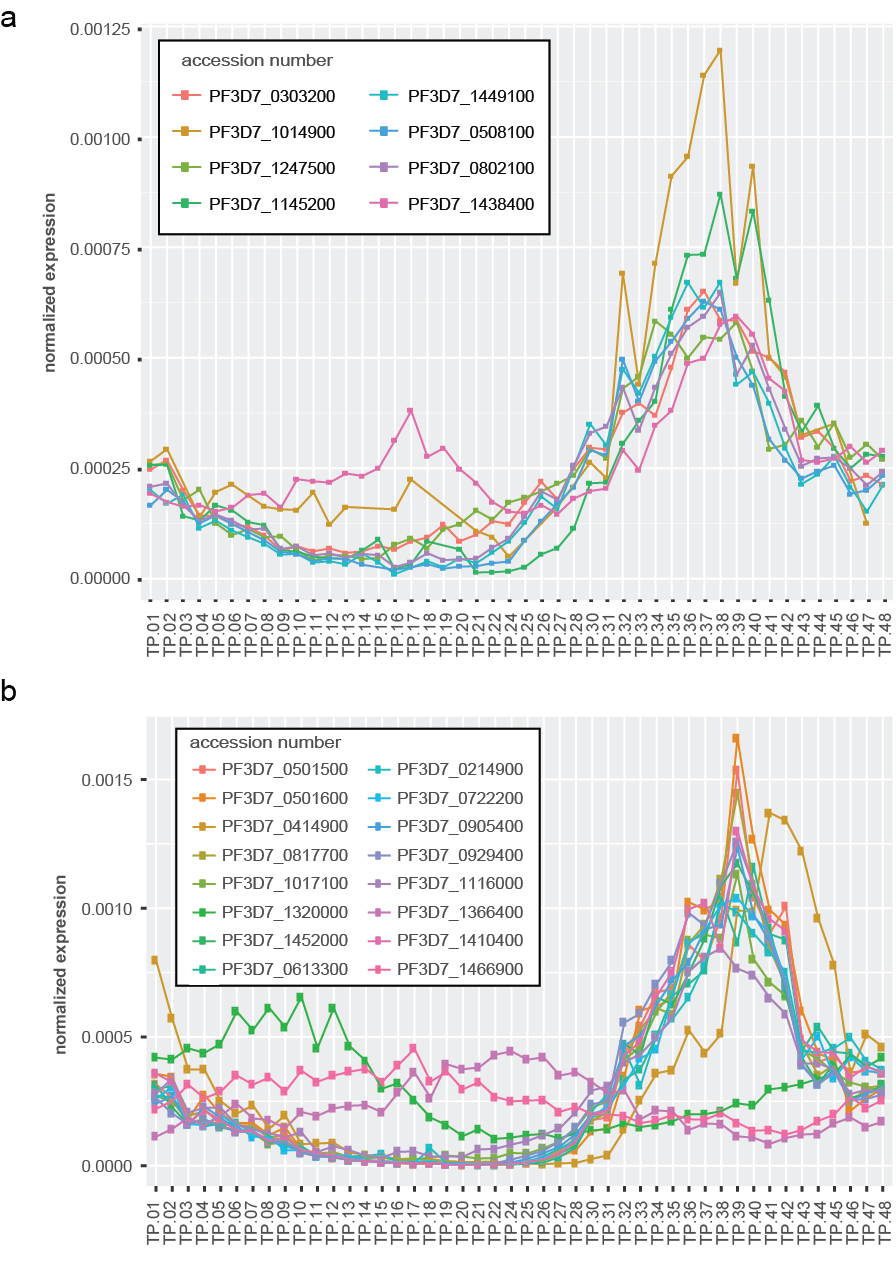

Supplement: Supplementary file 4 [file wellcomeopenres-3-16217-s0003.tgz › b7eb7571-e731-4e75-99ed-0e98429870f3.png]

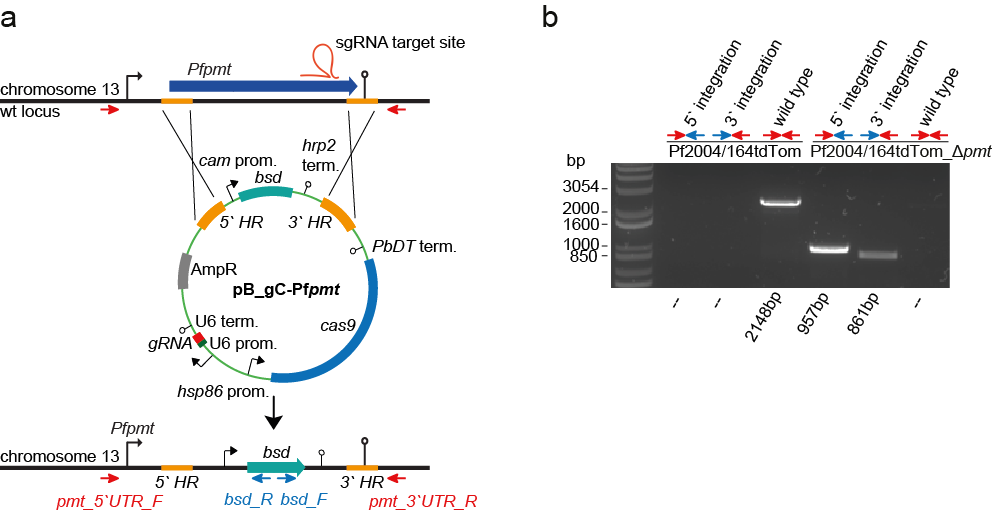

Supplement: Supplementary file 5 [file wellcomeopenres-3-16217-s0004.tgz › c0164682-11f9-4b1b-b8ee-980e45a5c725.png]

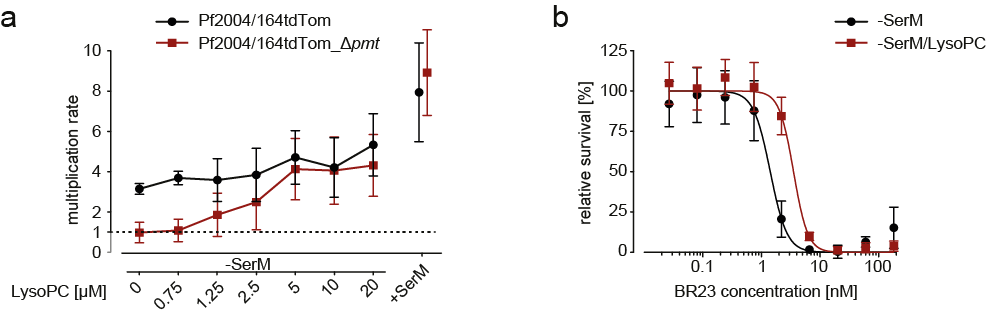

Supplement: Supplementary file 6 [file wellcomeopenres-3-16217-s0005.tgz › 51c14feb-4667-41d6-bd3b-f2a6c5479309.png]
