# Supplementary material for: Probing Plasmodium falciparum sexual commitment at the single-cell level
Source: Wellcome Open Res. 2018 Oct 17;3:70. Originally published 2018 Jun 13. [Version 4] doi: 10.12688/wellcomeopenres.14645.4 (PMC6143928; doi:10.12688/wellcomeopenres.14645.4)
Supplement: Supplementary file 12 [file wellcomeopenres-3-16217-s0011.tgz › 57f271b6-fd1c-4aad-93f3-56003ca7f6c0.docx]

| **Name** | **Direction** | **Sequence (5’-3’)** |
| --- | --- | --- |
| sgRNA_*pmt_*F | F | ATATTAAGTATATAATATTATATTCTTCAACAGTTATTAGTTTTAGAGCTAGAA |
| sgRNA_*pmt*_R | R | TTCTAGCTCTAAAACTAATAACTGTTGAAGAATATAATATTATATACTTAATAT |
| *pmt*_HR1_F | **F** | **GAGGAAGCGGAAGCTT**TAGAATCCTGTTTTGTTCCC |
| *pmt*_HR1_R | **R** | **TTGTGGATCCGAATTC**GGTAGCTTCCAAACCTC |
| *pmt* _HR2_F | **F** | **ATTTATTAAACTCGAG**GTCTTGATGATGGCTGG |
| *pmt*_HR2_R | **R** | **TTCAGGGTAGCTCGAG**ACACAAACATACGCATGG |
| *pmt*_5’UTR_F | F | TCATTAAAATTGTTGAATGG |
| BSD_5’UTR_R | R | TGTTTCTGCCTTTTCTC |
| BSD_3’UTR_F | F | TAATTTATGGGATAGCG |
| *pmt*_3’UTR_R | R | TATATATTACTGAATTTTACTC |
|  |  |  |
