# Supplementary material for: Probing Plasmodium falciparum sexual commitment at the single-cell level
Source: Wellcome Open Res. 2018 Oct 17;3:70. Originally published 2018 Jun 13. [Version 4] doi: 10.12688/wellcomeopenres.14645.4 (PMC6143928; doi:10.12688/wellcomeopenres.14645.4)
Supplement: Peer review history

# Peer review: Referee response for version 3

**DOI:** 10.21956/wellcomeopenres.16113.r33800

**Published:** 2018-09-17

**Authors:** Björn F.C. Kafsack

**Affiliations:** 1 Department of Microbiology and Immunology, Weill Cornell Medicine, New York City, NY, USA

The authors have address my remaining concerns. All I would ask for is the inclusion of higher resolution images with improved color balance for the Giemsa stains associated with Figure 4a.

I have read this submission. I believe that I have an appropriate level of expertise to confirm that it is of an acceptable scientific standard.

# Peer review: Referee response for version 2

**DOI:** 10.21956/wellcomeopenres.16028.r33579

**Published:** 2018-08-21

**Authors:** Björn F.C. Kafsack, Asaf Poran

**Affiliations:** 1 Department of Microbiology and Immunology, Weill Cornell Medicine, New York City, NY, USA; 2 Weill Cornell Medical College, New York, NY, USA

The initial version of the manuscript raised two major concerns with respect to (1) quality filtering of the single cell sequencing data and (2) regarding the quantification of sexual commitment in the Pf2004/164dtTom_∆pmt. This revised manuscript fully removes our concerns regarding the former but unfortunately left the latter unaddressed.

Quality Filtering

 Our issues regarding QC were based the use of “unique reads” to indicated UMIs since a single transcript with a single UMI can give rise to hundreds of unique (non-identical) reads because the tagmentation step occurs following cDNA PCR amplification. Since the QC threshold was actually 300 UMIs rather than 300 unique reads, concerns regarding the data quality are resolved. To improve clarity, “unique reads” should be replaced by “UMIs” or “individual transcripts”, particularly in the description of quality filtering.

Quantification of sexual commitment in the Pf2004/164dtTom_∆pmt line.

 I am unclear how providing the actual data underlying the normalized values being presented in Figure 4A could be considered beyond the scope of this manuscript. The answers to these questions seem integral to the interpretation of the data presented.

 Without providing the actual gametocyte frequencies along-side the normalized counts it would be impossible to assess whether loss of *pmt* results in change in commitment at all and how well suppression of this can be determined under various LysoPC concentrations. This is important since the results in Bobenchick et al. show a reduction the pmt∆ line at all time points. It is therefore not possible to discern whether the pmt∆ line is defective solely in gametocyte maturation or both commitment and maturation.

 The results presented Fig. 4A of this manuscript are normalized within each strain to the commitment level at 0uM LysoPC. But since the Pf2004/164dtTom_∆pmt line seems unable to replicate (the replication rate is 1,ie no increase in parasitemia) under those conditions, it begs the questions what that 100% value represents. Are the Pf2004/164dtTom_∆pmt parasite converting into gametocytes without replication under Serum-free conditions or does each schizonts only yield a single invasion event on average? To support the author’s claim that PMT in not required for the repression of commitment by LysoPC, it is important to understand what actually being repressed if Pf2004/164dtTom_∆pmt do not replicate and gametocytes do not develop under these conditions.

 Additionally, if under serum/LysoPC-free conditions Pf2004/164dtTom_∆pmt gametocytes don’t develop as stated by the authors, providing visual examples of Pf2004/164dtTom_∆pmt gametocytes under these conditions is critical to know what is actually being counted particularly since these cells provide the reference value for all LysoPC concentrations in the Pf2004/164dtTom_∆pmt line.

 All our other points were been sufficiently addressed.

We have read this submission. We believe that we have an appropriate level of expertise to confirm that it is of an acceptable scientific standard, however we have significant reservations, as outlined above.

# Response

**Published:** 2018-08-27

**Authors:** Matthias Marti

**Affiliations:** University of Glasgow, UK

Quality Filtering

 Our issues regarding QC were based the use of “unique reads” to indicated UMIs since a single transcript with a single UMI can give rise to hundreds of unique (non-identical) reads because the tagmentation step occurs following cDNA PCR amplification. Since the QC threshold was actually 300 UMIs rather than 300 unique reads, concerns regarding the data quality are resolved. To improve clarity, “unique reads” should be replaced by “UMIs” or “individual transcripts”, particularly in the description of quality filtering.

**We have clarified throughout the manuscript that analyses are based on UMIs rather than unique reads.**

Quantification of sexual commitment in the Pf2004/164dtTom_∆pmt line.

 I am unclear how providing the actual data underlying the normalized values being presented in Figure 4A could be considered beyond the scope of this manuscript. The answers to these questions seem integral to the interpretation of the data presented.

 Without providing the actual gametocyte frequencies along-side the normalized counts it would be impossible to assess whether loss of *pmt* results in change in commitment at all and how well suppression of this can be determined under various LysoPC concentrations. This is important since the results in Bobenchick et al. show a reduction the pmt∆ line at all time points. It is therefore not possible to discern whether the pmt∆ line is defective solely in gametocyte maturation or both commitment and maturation.

 The results presented Fig. 4A of this manuscript are normalized within each strain to the commitment level at 0uM LysoPC. But since the Pf2004/164dtTom_∆pmt line seems unable to replicate (the replication rate is 1,ie no increase in parasitemia) under those conditions, it begs the questions what that 100% value represents. Are the Pf2004/164dtTom_∆pmt parasite converting into gametocytes without replication under Serum-free conditions or does each schizonts only yield a single invasion event on average? To support the author’s claim that PMT in not required for the repression of commitment by LysoPC, it is important to understand what actually being repressed if Pf2004/164dtTom_∆pmt do not replicate and gametocytes do not develop under these conditions.

**We have revised Figure 4a and now show absolute conversion rates rather than normalized rates. As mentioned in the figure legend reduced PMR in the PMT KO under limited or no LysoPC conditions demonstrates that PMT is essential under these conditions. But conversion rates are titratable down to a PMR of 1 (hence, at 12% conversion rate every 12 ^th^ surviving schizont produces gametocyte progeny). Therefore, PMT activity is not required for sexual commitment.**

 Additionally, if under serum/LysoPC-free conditions Pf2004/164dtTom_∆pmt gametocytes don’t develop as stated by the authors, providing visual examples of Pf2004/164dtTom_∆pmt gametocytes under these conditions is critical to know what is actually being counted particularly since these cells provide the reference value for all LysoPC concentrations in the Pf2004/164dtTom_∆pmt line.

**We have also included Giemsa smears from WT and KO parasites at the time point of gametocyte readout (Stage II), at 0** **μM LysoPC** **. There is no apparent difference in morphology suggesting gametocyte development is not affected in the KO until Stage II. The figure legend has been modified accordingly.**

**However, we agree that gametocyte viability is likely reduced at Stage II (i.e., our time point of readout) in the PMT KO, as effects on later development and transmission have been observed (Pessi *et al*., PNAS 2004). As conversion rates are unchanged compared to WT even in complete absence of LysoPC, we conclude that both asexual and sexual stages are equally affected in the PMT KO.**

# Peer review: Referee response for version 1

**DOI:** 10.21956/wellcomeopenres.15947.r33322

**Published:** 2018-07-09

**Authors:** Björn F.C. Kafsack, Asaf Poran

**Affiliations:** 1 Department of Microbiology and Immunology, Weill Cornell Medicine, New York City, NY, USA; 2 Weill Cornell Medical College, New York, NY, USA

In their recent study in cell, the authors convincingly showed that sexual conversion in P. falciparum is higher responsive to availability of the serum metabolite LysoPC.

 In this they use LysoPC depletion to explore the explore transcriptional heterogeneity related to sexual commitment at the single cell level using a single-cell RNA sequencing approach.Single cells from three time points during schizogony are collected and sequenced, both under inducing and uninducing conditions.

 Relatively to previously published single-cell studies, the author detect low numbers of UMI / genes, with respect to the high sequencing depth. This low depth leads to weak association with bulk RNA seq data. The authors claim to define a gene signature specific to sexual commitment (10 genes), first through clustering of their data using SC3, following by a correlation analysis (125 genes).

 The authors then use AP2-G GFP and microscopy of single-cells in single wells to track and quantify fate-determination. Using this approach, the authors are able to confirm that the merozoites from a single schizonts are either all asexually- or sexually- committed, without mixing. The authors show that LysoPC depletion (-SerM conditions) both increases sexual commitment and reduces the number of merozoites progeny.

 The authors use flow cytometry and microscopy to validate some of the single-cell transcriptional analysis, such as the upregulation of PMT and EK enzymes in all parasites under LysoPC depletion, regardless of their sexual commitment. The authors also show that as predicted by transcriptomics, expression of rhoptry genes is altered in sexually committed parasites.

**Major Concerns:**

**1. Quality of single cell transcriptomes**

 Comparing the results of this study to the two other published scRNA-seq studies of malaria blood stages (Poran et al., Reid et al.) shows that the DGE scRNA-seq platform used yields single cell transcriptomes of substantially lower quality across a variety of measures.

                                                 This study        Reid et al.        Poran et al.

 Sequencing depth (read/cell) ~1e6                ~1-3e6             8e4

 Uniquely mapped reads         15-36%            75%                 >80%

 UMI/reads from rRNA             95-97%            17-34%            <5%                

 Unique mRNA genes-

 detected/cell/50k reads           20-50               184                  352

 Cells passing QC                    88%                 80-90%            82%

                        

 While the fraction of cells passing quality control is similar in all three studies, despite similar sequencing depths, the QC threshold used in this study (>300 unique reads/cell) is nearly two orders of magnitude lower than Reid et al. (25,000 reads/cell & >1000 genes detected).

 It is also much lower than the (>300 UMI/cell, about 20,000 reads per cell) our study.

 The technical quality of the single cell data raises significant concerns to the reproducibility of the single cell analyses.

 Given the high cost (nearly $35,000 = $30/cell * 384 cells/tp * 3 time-points) for the analysis of approximately 1000 cells, the claim that DGE is "cost-effective & high-throughput" should be qualified, in light of the available alternative approaches.

 This might suggest a substantial problem with the quality of collected cells (e.g. effect of sorting or Vibrant Violet staining), the effect of cryo-preservation, or library preparation.

 The resulting transcriptional noise is reflected by the very low correlation with bulk RNAseq collected under the same conditions (R^2=0.1-0.15). In light of such weak correlation, the claim that these single cell profiles "recapitulate overall transcriptional profiles from population-level RNA-seq experiments" should be reevaluated.

 The study by Reid et al, eLife 2018 also used a well-based method and sequenced to similar depths, and includes optimization of the library generation process that resolves many of the issue noted above. Given the similarities in approach and problems encountered, the lack of citation or discussion of the Reid et al. study is a critical gap in this manuscript.

**2. Lack of commitment phenotype in Pf2004/164tdTom_∆pmt**

 To test whether the given upregulation of *pmt* under LysoPC-deprivation is required for sexual conversion, ∆pmt was deleted in the Pf2004/164tdTom background. Figure 4a shows that while loss of *pmt* had an impact on the parasite’s ability to grow under low LysoPC concentrations, no impact on sexual conversion was observed.

 This finding is quite surprising given the study by Bobenchik et al. (PNAS 2013, Fig 2) that gametocyte formation was drastically reduced in PMT knockout parasites and that this phenotype could be fully restored to WT levels by genetic complementation of pmt. This study should be referenced and possible explanations of these differences should be discussed.

 Are the ∆pmt parasite still forming GCs despite being unable to replicate in the absence of LysoPC as indicated? Please provide actual % gametocytemia in addition to relative gametocytemia would help answer this question. As would images to validate the gametocyte morphology of ∆pmt tdTomato positive cells under these conditions

 In the interpretation of figure 4, the text states "In support of these data, imaging flow cytometry also revealed increased protein expression of the second enzyme involved in producing choline from the alternative substrate ethanolamine (ethanolamine kinase, ek) in all cells under −SerM conditions"

 Are the authors suggesting that that upregulations of EK can compensate for loss of PMT?

 EK converts ethanolamine into phospho-ethanolamine, which is converted into phospho-choline by PMT.

**Additional Notes:**

 Fig 2a:

 Indicating the expression of ap2-g in all 3 time points would be useful.

 Figure 2b

 Bars shown in the left panel represent two completely different readouts (%ap2-g+c for single cell & FPKM for bulk) and should not be place side by side in the same panel.

 Also, the text states that "Both analyses demonstrate that ap2-g expression is significantly increased over time (Figure 2b)" but Fig. 2b has no time component.

 Fig 2c:

 "40-48 hpi revealed robust and reproducible separation of individual cells into 12 clusters"

 9/12 clusters appear to have a stability index of 0.1. Clusters 5 and 11 appear to be the only stable clusters. The heatmap strongly suggests that Cluster 9 is composed of 2 clusters.

 The text also states that "Expression of 10 genes, including ap2-g and set9, defined a subset of cells (cluster 11)" but 2c appears to show that set9 was detected in the majority of cells regardless of cluster.

 Why are cells with >80%ile pmt expression indicated when the text states that *pmt* and *ek* “were equally expressed across all cells” and the fact the scRNAseq is quite poor at making quantitative comparisons?

 Fig 2d:

 Moreover, the data in Fig 2d seem to contradict the earlier statement that *pmt* and *ek* aren’t equally expressed across the clusters but rather lower in clusters with high “signature” scores.

 The text state that "Expression of ap2-g and msrp1 is significantly enriched in the clusters highlighted in red, while expression of pmt and ek are not" While PMT and EK are not higher in the clusters, they do appear lower particularly for clusters 10-11.

 For clarity, color coding of significant difference (higher or lower) of mean gene expression in these clusters should be done individually by gene/cluster rather than for all 4 genes in a column.

 What fraction of the variation captured by the signature is just the serine/threonine kinase PF3D7_1145200? The heatmap would suggest it’s a or, possibly, the major component.

 Providing gene names when available in the heatmap portion would help interpretation.

 The upstream regions of “commitment signature” gene identified in Poran et al. were substantially enrich in AP2-G binding sites. A similar analysis would be a valuable addition to help evaluate whether differences in signature genes in this study may be upstream or downstream of ap2-g expression. 

 Fig 2e.

 Please clarify the ratio being calculated on the x-axis. The legend states:

 "Single cells from 40-48 hpi separate into subsets with high and low marker signature (above and below the 80th percentile, respectively), with 125 differentially expressed genes measured by scRNA-seq, including ap2-g, msrp1 and set9."

 Does that mean that the fold-change being calculated is between the top 20% of signature expressing cells vs the rest? That seems unlikely as the argument would then become circular.

 Since AP2-G expression is the current standard for commitment, plotting the ratio of AP2-G+ vs AP2-G- cells may be the most appropriate. Please indicate in the legend if the plotted p-values were Benjamini-Hochberg adjusted. 

 Fig 4d

 The number of cells analyzed should be indicated.

 Fig 4e

 The legend indicates that all three compartments include the “iRBC membrane”. I suspect this is in error as compartment should be mutually exclusive to sum to 100%.

 The number of cells analyzed should be indicated.

 The methods are unclear as to how these compartments were defined in the absence of additional markers for the RBC PM or PVM.

 The text seems to indicate that the microscopy results in fig 4e confirms upregulation of RhopH3. However, the last time-point shows no differences levels of RhopH3, suggests that RhopH3 expression may begin earlier in AP2-G+ cells but that expression in AP2-G- cells catches up yielding similar RhopH3 distribution in mature AP2-G+ and AP2-G- schizonts, as shown.

We have read this submission. We believe that we have an appropriate level of expertise to confirm that it is of an acceptable scientific standard, however we have significant reservations, as outlined above.

# Response

**Published:** 2018-07-23

**Authors:** Matthias Marti

**Affiliations:** University of Glasgow, UK

**Major Concerns:**

**1. Quality of single cell transcriptomes**

 Comparing the results of this study to the two other published scRNA-seq studies of malaria blood stages (Poran et al., Reid et al.) shows that the DGE scRNA-seq platform used yields single cell transcriptomes of substantially lower quality across a variety of measures.

                                                 This study        Reid et al.        Poran et al.

 Sequencing depth (read/cell) ~1e6                ~1-3e6             8e4

 Uniquely mapped reads         15-36%            75%                 >80%

 UMI/reads from rRNA             95-97%            17-34%            <5%                

 Unique mRNA genes-

 detected/cell/50k reads           20-50               184                  352

 Cells passing QC                    88%                 80-90%            82%

                        

 While the fraction of cells passing quality control is similar in all three studies, despite similar sequencing depths, the QC threshold used in this study (>300 unique reads/cell) is nearly two orders of magnitude lower than Reid et al. (25,000 reads/cell & >1000 genes detected).

 It is also much lower than the (>300 UMI/cell, about 20,000 reads per cell) our study.

 The technical quality of the single cell data raises significant concerns to the reproducibility of the single cell analyses.

**Our QC threshold was 300 UMI/cell, regardless of the number of total reads coming from that cell. We acknowledge that our data were produced less efficiently due to poorer rDNA depletion, which could be improved if the method were implemented in the future. Nevertheless, we feel that the biological results are reproducible. Please note below our analysis of enriched AP2-G binding sites in genes that are positively differentially expressed in our list of loci associated with sexual commitment signature.**

 Given the high cost (nearly $35,000 = $30/cell * 384 cells/tp * 3 time-points) for the analysis of approximately 1000 cells, the claim that DGE is "cost-effective & high-throughput" should be qualified, in light of the available alternative approaches.

**We have moderated language regarding cost-effectiveness. We do note that the protocol has limited hardware requirements relative to 10X or DropSeq.**

 This might suggest a substantial problem with the quality of collected cells (e.g. effect of sorting or Vibrant Violet staining), the effect of cryo-preservation, or library preparation.

**Please note that cells have not been cryopreserved prior to sorting.**

 The resulting transcriptional noise is reflected by the very low correlation with bulk RNAseq collected under the same conditions (R^2=0.1-0.15). In light of such weak correlation, the claim that these single cell profiles "recapitulate overall transcriptional profiles from population-level RNA-seq experiments" should be reevaluated.

**There is a significant, albeit weak, positive correlation, indicating that overall, there is quite a bit of noise from the single cell data, but the general trends from the bulk RNA-seq hold. We have moderated the language discussing this result and have removed Figure 1f.**

 The study by Reid et al, eLife 2018 also used a well-based method and sequenced to similar depths, and includes optimization of the library generation process that resolves many of the issue noted above. Given the similarities in approach and problems encountered, the lack of citation or discussion of the Reid et al. study is a critical gap in this manuscript.

**Thank you for your suggestion. We have added Reid *et al.*to the references.**

**2. Lack of commitment phenotype in Pf2004/164tdTom_∆pmt**

 To test whether the given upregulation of *pmt* under LysoPC-deprivation is required for sexual conversion, ∆pmt was deleted in the Pf2004/164tdTom background. Figure 4a shows that while loss of *pmt* had an impact on the parasite’s ability to grow under low LysoPC concentrations, no impact on sexual conversion was observed.

 This finding is quite surprising given the study by Bobenchik et al. (PNAS 2013, Fig 2) that gametocyte formation was drastically reduced in PMT knockout parasites and that this phenotype could be fully restored to WT levels by genetic complementation of pmt. This study should be referenced and possible explanations of these differences should be discussed.

**Indeed, our data demonstrate that PMT is not required to repress sexual commitment. However, it is required for gametocyte development and maturation as shown by Bobenchik et al. This is also supported by our own preliminary experiments.**

**We have added the Bobenchik *et al.*reference in the discussion.**

 Are the ∆pmt parasite still forming GCs despite being unable to replicate in the absence of LysoPC as indicated? Please provide actual % gametocytemia in addition to relative gametocytemia would help answer this question. As would images to validate the gametocyte morphology of ∆pmt tdTomato positive cells under these conditions.

**This is an interesting question and subject of ongoing studies in our lab, but outside of the scope of this MS.**

 In the interpretation of figure 4, the text states "In support of these data, imaging flow cytometry also revealed increased protein expression of the second enzyme involved in producing choline from the alternative substrate ethanolamine (ethanolamine kinase, ek) in all cells under −SerM conditions"

 Are the authors suggesting that that upregulations of EK can compensate for loss of PMT?

 EK converts ethanolamine into phospho-ethanolamine, which is converted into phospho-choline by PMT.

**It does not refer to the PMT KO data but the -SerM conditions of WT Pf2004/164tdTom parasites, as shown in Figure 4b and referenced in the text accordingly.**

**Additional Notes:**

 Fig 2a:

 Indicating the expression of ap2-g in all 3 time points would be useful.

**This is a good suggestion - we have now marked *ap2-g*across the three time points.**

 Figure 2b

 Bars shown in the left panel represent two completely different readouts (%ap2-g+c for single cell & FPKM for bulk) and should not be place side by side in the same panel.

 Also, the text states that "Both analyses demonstrate that ap2-g expression is significantly increased over time (Figure 2b)" but Fig. 2b has no time component.

**Thank you for catching this error. We agree that it may be misleading and will adjust the figure accordingly. We have updated the text with a more accurate description of panel b.**

 Fig 2c:

 "40-48 hpi revealed robust and reproducible separation of individual cells into 12 clusters"

 9/12 clusters appear to have a stability index of 0.1. Clusters 5 and 11 appear to be the only stable clusters. The heatmap strongly suggests that Cluster 9 is composed of 2 clusters.

**Altogether, these issues may derive from the complex and incomplete nature of single-cell transcriptomes.** **A lower stability index indicates that upon choosing either 11 or 13 clusters, these cells were not entirely clustered together, which indicates how robust a cluster is to the a priori number of clusters. We acknowledge there may be some strong sub-clustering, especially within cluster 9 as you mentioned. We opted for 12 clusters as this was estimated by the Tracy-Widom theory to be the optimal number of clusters. We could obviously choose a larger number of clusters to have “stronger” clusters, but at the cost of potentially splitting up relevant and real clusters of cells.** 

 The text also states that "Expression of 10 genes, including ap2-g and set9, defined a subset of cells (cluster 11)" but 2c appears to show that set9 was detected in the majority of cells regardless of cluster.

**Set9 was detected in a majority of cells, but *set9*expression was elevated in cluster 11. We have adjusted the text to be more clear.**

 Why are cells with >80%ile pmt expression indicated when the text states that *pmt* and *ek* “were equally expressed across all cells” and the fact the scRNAseq is quite poor at making quantitative comparisons?

**We agree that the presentation of *pmt*in Figure 2c is potentially confusing, given that figure 2d much more clearly and quantitatively illustrates the expression level of *pmt*and *ek*in the cells comprising each cluster. We have removed *pmt*and the other genes previously displayed on the top rows of Figure 2c, and have amended the text accordingly.**

 Fig 2d:

 Moreover, the data in Fig 2d seem to contradict the earlier statement that *pmt* and *ek* aren’t equally expressed across the clusters but rather lower in clusters with high “signature” scores.

 The text state that "Expression of ap2-g and msrp1 is significantly enriched in the clusters highlighted in red, while expression of pmt and ek are not" While PMT and EK are not higher in the clusters, they do appear lower particularly for clusters 10-11. 

**The difference is not statistically significant.**

 For clarity, color coding of significant difference (higher or lower) of mean gene expression in these clusters should be done individually by gene/cluster rather than for all 4 genes in a column.

**These colors indicated that the signature was elevated, not the individual gene expressions. We have attempted to clarify this in the figure and legend.** 

 What fraction of the variation captured by the signature is just the serine/threonine kinase PF3D7_1145200? The heatmap would suggest it’s a or, possibly, the major component.

**The signature used normalized expression values such that highly expressed genes are not weighed more than lower genes. As referee 1 pointed out, the scale in panel d does not clearly illustrate expression level differences, and we have attempted to rectify this with a different color map.**

 Providing gene names when available in the heatmap portion would help interpretation.

**We have done so.** 

 The upstream regions of “commitment signature” gene identified in Poran et al. were substantially enrich in AP2-G binding sites. A similar analysis would be a valuable addition to help evaluate whether differences in signature genes in this study may be upstream or downstream of ap2-g expression. 

**Thank you for this suggestion. We have performed this analysis using predicted AP2-G binding motifs from Campbell *et al.*2010, which identified such motifs up to 2 kb upstream of translational start sites. Overall, 84% of the genes we detected with our DGE data had AP2-G binding sites, but 94% (117/125) of the genes we identified as being positively differentially expressed in our commitment signature gene set harbored an AP2-G binding site, a significant enrichment (Fishers exact test, P=0.002) that could indicate a significant proportion of the loci we detected are expressed downstream of AP2-G activation. We have included this new analysis result in the text.**

 Fig 2e.

 Please clarify the ratio being calculated on the x-axis. The legend states:

 "Single cells from 40-48 hpi separate into subsets with high and low marker signature (above and below the 80th percentile, respectively), with 125 differentially expressed genes measured by scRNA-seq, including ap2-g, msrp1 and set9."

 Does that mean that the fold-change being calculated is between the top 20% of signature expressing cells vs the rest? That seems unlikely as the argument would then become circular.

 Since AP2-G expression is the current standard for commitment, plotting the ratio of AP2-G+ vs AP2-G- cells may be the most appropriate. Please indicate in the legend if the plotted p-values were Benjamini-Hochberg adjusted. 

**We have clarified the ratio in the text. We used bulk-verified genes to create the marker signature, and show results from those in c and d. In e, we used all genes detected in single cell. We did expect the genes used to create the signature to appear in this analysis, but we also wanted to look for potentially other genes that were not picked up by the bulk analysis.**

**The p-values were corrected and we have more clearly denoted this.** 

 Fig 4d

 The number of cells analyzed should be indicated. 

**The number of cells analysed was 100 per time point, per condition. This is now denoted in the figure legend.**

 Fig 4e

 The legend indicates that all three compartments include the “iRBC membrane”. I suspect this is in error as compartment should be mutually exclusive to sum to 100%. 

**“iRBC membrane” is correct. The iRBC membrane staining remains constant as it reflects RhopH3 localization from the previous cycle. Therefore, the compartments where labeling changes in each condition are the parasite membrane, the iRBC cytosol, or the Rhoptries.**

 The number of cells analyzed should be indicated. 

**The number of cells analysed in all our work was 100 per time point, per condition. We have included this in the figure legend as well.**

 The methods are unclear as to how these compartments were defined in the absence of additional markers for the RBC PM or PVM. 

**These compartments are a proxy of maturation and/or commitment, and not a detailed co-localization analysis. Using DIC, bright field and fluorescence microscopy, it is possible to define the parasite and the RBC boundaries, as well as the RBC cytosol. By fluorescence intensity it is possible to define the rhoptries and distinguish them from other types of signal arising from more disperse presence. The image quality and resolution we obtained are comparable to those of other publications using the same antibodies.**

 The text seems to indicate that the microscopy results in fig 4e confirms upregulation of RhopH3. However, the last time-point shows no differences levels of RhopH3, suggests that RhopH3 expression may begin earlier in AP2-G+ cells but that expression in AP2-G- cells catches up yielding similar RhopH3 distribution in mature AP2-G+ and AP2-G- schizonts, as shown. 

**The data in 4d demonstrate that de novo rhoptry gene transcription (based on normalized mRNA expression of all rhoptry-related genes) and rhoptry protein synthesis (based on RhopH3 mean fluorescence intensity) is increased across time points in AP2-G+ cells compared to AP2-G- cells, suggesting overall increased levels rather than just a slight shift in cell cycle in AP2-G+ cells. This difference is only partially explained by rhoptry signal intensity of RhopH3, however, as pointed out by this reviewer: rhoptry signal intensity (RhopH3) is only increased in the second time point in AP2-G+ compared to AP2G- cells. These were our observations in multiple repeated experiments, suggesting different dynamics in committed and non-committed parasites. As parasites reach full maturation at the schizont stage, this difference disappears. While there is no mechanistic observation on the reason for this, the observation was consistent and we considered it appropriate to report as such.**

# Peer review: Referee response for version 1

**DOI:** 10.21956/wellcomeopenres.15947.r33320

**Published:** 2018-07-04

**Authors:** Lyn-Marie Birkholtz, Riette van Biljon, Roelof van Wyk

**Affiliations:** 1 Malaria Parasite Molecular Laboratory, Department of Biochemistry, Institute for Sustainable Malaria Control, Medical Research Council Collaborating Centre for Malaria Research, University of Pretoria, Pretoria, South Africa; 2 University of Pretoria, Pretoria, South Africa

The ability of malaria parasites to differentiate into sexual forms is the basis for continued survival and propagation of disease. The paper by Brancucci et al., provides a new dataset describing commitment to gametocytogenesis by evaluating the transcriptional signature in sexually committed cells on a single-cell level. The strength of the paper lies in the identification of signatures associated only with differentiation, within a background of general metabolic and life cycle changes, and this was mediated by the development of a digital gene expression (DGE) platform. The authors argued that this allowed the heterogeneity of parasite populations to be captured on a transcriptional level.

 The authors combine their previous system, constricting lysoPC as environmental sensor, to produce gametocytes and subsequently capture individual committed cells to evaluate transcriptional signatures, confirmed through imaging. The transcriptional signatures are compared with bulk transcriptome datasets previously produced by these authors ^1^ and others (Bozdech *et al.* ^2^), but significantly also with previous single-cell sequencing data from committed parasites (Poran *et al.* ^3^). The work presented here by Brancucci correlates with their previous findings on bulk parasite cultures with similar experimental designs. This iterates that the observations made from bulk parasite cultures are indeed associated with commitment as delineated and confirmed by the scRNA-seq data presented here. Additionally, the manuscript provides information on genes involved in the processes preceding activation of the commitment master switch, AP2-G, which was not evaluated in the Poran study.

 The paper concludes with functional evaluation of the involvement of *pmt* in sexual commitment, concluding that activation thereof is independent of sexual commitment under lysoPC constriction. This leads to the postulation of a threshold-dependent regulation of lysoPC activation of commitment, and interesting proposal worthy of further evaluation.

 The paper therefore presents a timely contribution of a dataset that will aid further explorations and validations of signatures associated with gametocyte commitment. With the DGE platform, the paper makes a powerful resource to the malaria research community.

 The manuscript is well written, the figures nicely conceived and presented and the methods appropriately reported. The quality of the experimental system (DGE) and data produced is very good, given the limitations on starting material within this system, and should be taken up by the community as platform for further investigations. I support indexing of this paper.

 Technical aspects that could be clarified include:

 Figure 1a: The explanation as to the experimental setup needs clarification, including cell lines used for the single-cell transcriptome study, as this is not clearly defined in the methodology section. To avoid confusion, please clearly indicate if the single-cell transcriptomics was performed on non-modified lines, the Pf2004/164dTom line or the *pmt* knockout line. It is also not clear from the figure or legend what the ‘green’ population for FACS sorting and microscopic evaluation refers to, besides indicating the populations tagged for DGE?

 Figure 1d: The platform is validated through transcriptional differences observed between time points and a statement that ‘single-cell transcriptomes generally recapitulate overall transcriptional profiles from population-level RNAseq experiments’. However, care should be taken in as to the extent that correlations can be used to support these conclusions. The data presented in Fig 1d, is used to justify a major conclusion from figure 1- that the scRNA profiling is able to discriminate 4 h of temporal development. However, the single correlation (at R ^2^ of 0.43) with the complete bulk transcriptome time course from Bozdech 2003 (only the top 100 genes) is not entirely convincing (should perhaps just be included in supplementary data?).

 Fig 1f. Poor correlations of 0.1 and 0.15 is observed for the top 100 most transcribed genes between the scRNA-seq data and population-level data (from Brancucci 2017), and the authors conclude this to be ‘significantly through weakly’ correlated.  However, such low correlations are almost indicative of no correlation, even though significant. Could the authors elaborate why such low correlation is seen between the scRNA-seq and their own population-seq data produced under the same experimental conditions, but a correlation of 0.4 is seen when compared with the Bozdech 2003 population-seq data? As the authors rightly state, this could be a reflection of the loss of genes in single-cell sequencing, and should be left at that.

 Fig 2a. This figure has some findings that could be really important that is only presented in the scRNA-seq dataset and lacking in the bulk RNA dataset. Genes with an induction score of 0, but with positive pseudobulk log _2_FC values are therefore only seen at the scRNA-seq levels and potentially lost or obscured in bulk datasets. Could the authors elaborate on these genes as potential novel aspects of their dataset?

 Fig 2b: The relevance of the additional FPKM axis is not clear? Please clarify in the figure legend.

 Fig 2c: The signature value is not properly explained.

 Fig 2d: The activity scale used is not sufficient to show genes that express, with white indicating high expression even though it appears mostly inactive. The use of a 5-colour system may be more appropriate or transformations that place the values in a more comparable range.

 Table 2. The comparison with the Poran 2017 data clearly highlights the genes that correlate, but also indicates that the data here identifies genes involved in the processes upstream of AP2-G activation. It would be important to highlight the numbers and individual genes involved in these processes, which currently are not clearly stipulated, but do present a novel finding of this study.

We have read this submission. We believe that we have an appropriate level of expertise to confirm that it is of an acceptable scientific standard.

## References

[1] BrancucciNMBGerdtJPWangCDe NizMPhilipNAdapaSRZhangMHitzENiederwieserIBoltrykSDLaffitteMCClarkMAGrüringCRavelDBlancke SoaresADemasABoppSRubio-RuizBConejo-GarciaAWirthDFGendaszewska-DarmachEDuraisinghMTAdamsJHVossTSWatersAPJiangRHYClardyJMartiM: Lysophosphatidylcholine Regulates Sexual Stage Differentiation in the Human Malaria Parasite Plasmodium falciparum. *Cell*.2017;171(7) : 10.1016/j.cell.2017.10.020 1532-1544.e15 10.1016/j.cell.2017.10.020 29129376PMC5733390

[2] BozdechZLlinásMPulliamBLWongEDZhuJDeRisiJL: The transcriptome of the intraerythrocytic developmental cycle of Plasmodium falciparum. *PLoS Biol*.2003;1(1) : 10.1371/journal.pbio.0000005 E5 10.1371/journal.pbio.0000005 12929205PMC176545

[3] PoranANötzelCAlyOMencia-TrinchantNHarrisCGuzmanMHassaneDElementoOKafsackB: Single-cell RNA sequencing reveals a signature of sexual commitment in malaria parasites. *Nature*.2017; 10.1038/nature24280 10.1038/nature24280 PMC605593529094698

# Response

**Published:** 2018-07-23

**Authors:** Matthias Marti

**Affiliations:** University of Glasgow, UK

Figure 1a: The explanation as to the experimental setup needs clarification, including cell lines used for the single-cell transcriptome study, as this is not clearly defined in the methodology section. To avoid confusion, please clearly indicate if the single-cell transcriptomics was performed on non-modified lines, the Pf2004/164dTom line or the *pmt* knockout line. It is also not clear from the figure or legend what the ‘green’ population for FACS sorting and microscopic evaluation refers to, besides indicating the populations tagged for DGE?

**The line used for flow sorting and scRNAseq is Pf2004/164tdTom, hence the same as used in the previous cell paper by Brancucci et al. This has been clarified in the methods section and the figure legend. The green population represents sexually committed cells. This has also been added to figure legend 1.**

 Figure 1d: The platform is validated through transcriptional differences observed between time points and a statement that ‘single-cell transcriptomes generally recapitulate overall transcriptional profiles from population-level RNAseq experiments’. However, care should be taken in as to the extent that correlations can be used to support these conclusions. The data presented in Fig 1d, is used to justify a major conclusion from figure 1- that the scRNA profiling is able to discriminate 4 h of temporal development. However, the single correlation (at R2 of 0.43) with the complete bulk transcriptome time course from Bozdech 2003 (only the top 100 genes) is not entirely convincing (should perhaps just be included in supplementary data?).

**The single cell data are much sparser than the bulk transcriptomics data, and gene dropout effects may lower the correlation. Using a standard linear regression will not be robust to gene dropout and the sparseness of these data, but nevertheless show a modest, significant correlation. We acknowledge that the plot illustrating the correlation between scRNA and bulk profiles is not compelling (Fig 1f) and have consequently removed it.**

 Fig 1f. Poor correlations of 0.1 and 0.15 is observed for the top 100 most transcribed genes between the scRNA-seq data and population-level data (from Brancucci 2017), and the authors conclude this to be ‘significantly through weakly’ correlated.  However, such low correlations are almost indicative of no correlation, even though significant. Could the authors elaborate why such low correlation is seen between the scRNA-seq and their own population-seq data produced under the same experimental conditions, but a correlation of 0.4 is seen when compared with the Bozdech 2003 population-seq data? As the authors rightly state, this could be a reflection of the loss of genes in single-cell sequencing, and should be left at that.

**Again, gene dropout is a likely culprit here for why the single cell data only weakly correlates with bulk transcriptomics data. We agree the significance is driven by numbers, rather than a strong correlation. However, in general, especially for the 100 most expressed genes, a higher relative expression in the population data led to a higher relative expression in the single cell data, which was our point with this panel.**

 Fig 2a. This figure has some findings that could be really important that is only presented in the scRNA-seq dataset and lacking in the bulk RNA dataset. Genes with an induction score of 0, but with positive pseudobulk log2FC values are therefore only seen at the scRNA-seq levels and potentially lost or obscured in bulk datasets. Could the authors elaborate on these genes as potential novel aspects of their dataset?

**Some of those genes were not significantly differentially expressed by single cell, but those that were include ETRAMP14, CLAG3.1, and RhopH3. After multiple hypothesis correction, only CLAG3.1 remains. The single-cell data were unbalanced, with more induced than uninduced cells; this, combined with gene dropout, may lead to some false discovery, though p-value correction would in theory overcome this issue. We instead chose to focus on genes that were corroborated by the bulk dataset, as we have higher confidence in those results than discordant genes.**

 Fig 2b: The relevance of the additional FPKM axis is not clear? Please clarify in the figure legend.

**FPKM is the axis for the population data. We acknowledge this is a confusing presentation and have separated the first plot in Fig 2b into two separate plots.**

 Fig 2c: The signature value is not properly explained.

**The signature value is explained in the methods, but was unfortunately omitted from the legend. We have now added it to the legend to correct this.**

 Fig 2d: The activity scale used is not sufficient to show genes that express, with white indicating high expression even though it appears mostly inactive. The use of a 5-colour system may be more appropriate or transformations that place the values in a more comparable range.

**Thank you for your suggestions. We adjusted the scale to improve clarity.**

 Table 2. The comparison with the Poran 2017 data clearly highlights the genes that correlate, but also indicates that the data here identifies genes involved in the processes upstream of AP2-G activation. It would be important to highlight the numbers and individual genes involved in these processes, which currently are not clearly stipulated, but do present a novel finding of this study.

**Please see our response below to REVIEWER 1, in which we have further investigated the genes which may be upstream vs. downstream of AP2-G activation through an analysis of AP2-G binding sites in promotor regions. We have included a more detailed description of the genes in the revised draft.**
